# WNT signaling in cancer: molecular mechanisms and potential therapies

**DOI:** 10.1186/s43556-025-00327-x

**Published:** 2025-10-22

**Authors:** Jiaqi Liang, Yiming Pan, Jingru Yang, Dequan Zeng, Jing Li

**Affiliations:** 1 State Key Laboratory of Oral Diseases & National Center for Stomatology & National Clinical Research Center for Oral Diseases & Research Unit of Oral Carcinogenesis and Management &, Chinese Academy of Medical Sciences, West China Hospital of Stomatology, Sichuan University, Chengdu, Sichuan 610041 China; 2 School of Health Management, Xihua University, Chengdu, 610041 China

**Keywords:** WNT Signaling, FZD, Cancer, Biomarker, Therapeutic targets, Inhibitor

## Abstract

The WNT signaling pathway, a fundamental molecular network regulating cell proliferation, differentiation, and stemness, plays a critical role in tumorigenesis, cancer progression, and therapeutic resistance. Given its crucial regulatory roles in tumors, WNT signaling pathway has been identified as effective targets for cancer treatment. However, the current clinical efficacy of WNT signaling pathway-targeted anti-tumor therapies remains suboptimal. Based on research investigating the role of WNT signaling pathway in cancer, we systematically discuss the molecular mechanisms of WNT signaling in cancer (including both canonical and non-canonical signaling pathways), the role of WNT signaling in different cancer types, highlighting distinct potential therapeutic approaches targeting WNT signaling. We also comprehensively review innovative strategies targeting WNT signaling, including Porcupine (PORCN) inhibitors, Tankyrase (TNKS) inhibitor, Frizzled (FZD)-targeted monoclonal antibodies, β-catenin/TCF transcriptional complex inhibitors, and natural bioactive compounds and drug repositioning etc., critically evaluating their preclinical efficacy and limitations. We emphasize the need for and challenges in developing WNT-targeted therapies including refining the specificity of WNT signaling pathway-targeted therapies, developing biomarkers for patient selection, and exploring synergies between WNT inhibitors and other therapeutic modalities such as immune checkpoint blockers. These advances aim to enable personalized precision therapy and revolutionize cancer treatment paradigms in the future.

## Introduction

The WNT signaling pathway is generally categorized into the canonical pathway and non-canonical pathways. As an evolutionarily conserved signaling pathway, the WNT pathway has been demonstrated to play pivotal roles in the development of multiple tissues and organs, including the skin, eyes, brain, lungs, spinal cord, teeth, bones, cartilage, kidneys, liver, hematopoietic system, and reproductive organs [1]. Furthermore, the WNT signaling pathway participates in the regulation of tissue homeostasis. For instance, the activation of WNT signaling is observed during tissue repair processes following hepatic and pulmonary injuries. This pathway regulates fundamental cellular behaviors including proliferation, differentiation, apoptosis, and migration [2]. Given the extensive physiological functions of WNT signaling, its dysregulation is closely associated with the pathogenesis of various diseases. An increasing number of studies indicate that aberrant WNT activation contributes to cancer-related mortality by promoting tumorigenesis, cancer cell proliferation, differentiation, metastasis, invasion, and therapeutic resistance [3]. Consequently, the WNT signaling pathway represents a promising therapeutic target in cancer treatment.

Currently, substantial research efforts have been devoted to developing targeted therapeutics against WNT signaling activation, with several agents undergoing clinical trials. However, despite demonstrating anti-cancer potential in preclinical studies, various WNT inhibitors have exhibited adverse effects in clinical trials, including bone fractures and gastrointestinal toxicity [4]. The primary challenges stem from off-target effects that disrupt physiological WNT signaling in normal tissues and crosstalk between the WNT pathway and other signaling pathways. Balancing therapeutic efficacy with safety profiles remains a critical challenge and research focus for future WNT-targeted therapies. Recent advancements in enhancing therapeutic safety have been reported, encompassing strategies such as improving WNT inhibitor specificity, combinatorial approaches with other pathway inhibitors, and integration with immunotherapies.

In summary, we propose to systematically review the molecular mechanisms of the WNT signaling pathway and its crosstalk with other signaling pathways in cancer. Furthermore, we consolidate the roles of dysregulated WNT signaling in various cancers and categorize distinct types of WNT inhibitors currently under preclinical investigation and in clinical trials. Finally, we highlight existing challenges in targeting the WNT pathway and discuss potential synergies with other therapeutic modalities. We also address the emerging potential of WNT signaling components as biomarkers for diagnosis, therapeutic guidance, and prognostic evaluation.

## Molecular mechanisms of WNT signaling in cancer

The molecular mechanisms of the WNT signaling pathway have been extensively studied. Aberrations in components of both canonical and non-canonical pathways have been demonstrated to be closely associated with cancer progression. By comprehensively reviewing the latest advances in WNT signaling mechanisms, we can gain deeper insights into its pivotal role in cancer pathogenesis. Furthermore, the WNT signaling pathway extensively crosstalks with other signaling cascades, and this interplay collectively promotes tumor development. Therefore, we provide an updated overview of WNT signaling mechanisms, systematically summarize the aberrations in WNT signaling contributing to cancer, and highlight the critical network interactions between the WNT pathway and other signaling cascades.

### The canonical signaling pathway in cancer

**Fig. 1:**
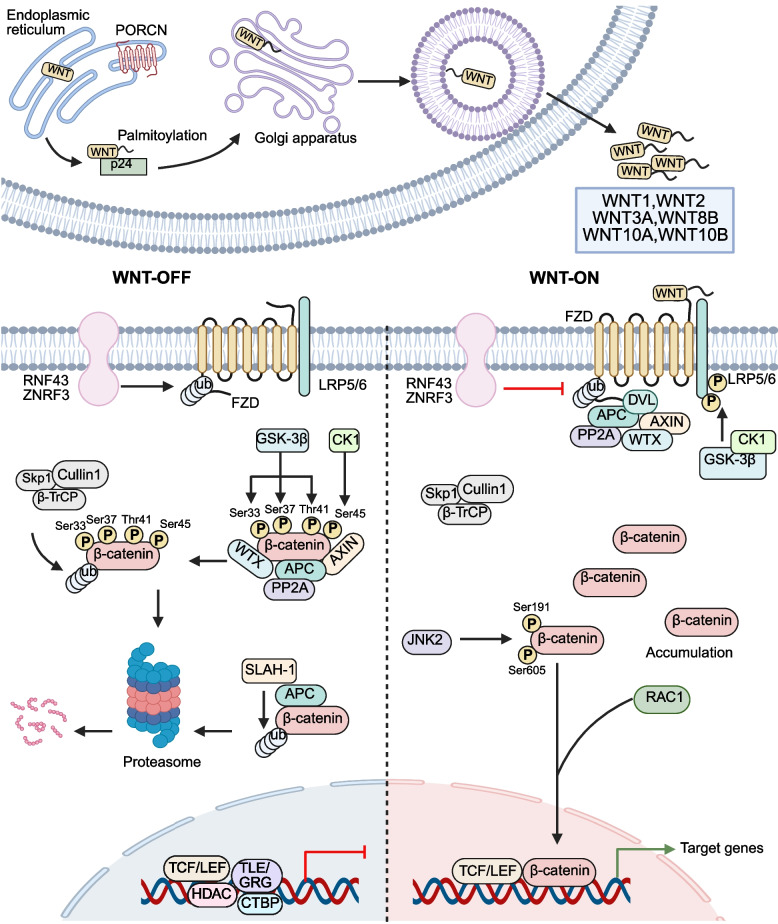
Signal transduction of the canonical WNT pathway. Under the canonical WNT signaling off conditions, the WNT ligand undergoes palmitoylation modification by PORCN and is secreted from the endoplasmic reticulum into the intracellular space. RNF43/ZNRF3 are actively expressed, ubiquitinating the FZD receptor to maintain low levels of WNT ligand stimulation. The destruction complex assembles and phosphorylates intracellular β-catenin, which is subsequently degraded by ubiquitin ligases such as β-TrCP. In contrast, during canonical WNT signaling activation, FZD is highly expressed and binds overexpressed WNT ligands. Upon receiving this signal, DVL is activated and inhibits the formation of the destruction complex. Meanwhile, CK1 and GSK-3β phosphorylate the co-receptor LRP5/6. As a result, β-catenin escapes phosphorylation by the destruction complex and is transported into the nucleus via the action of proteins such as JNK2 and RAC1. Once in the nucleus, β-catenin binds to TCF/LEF transcription factors, initiating the transcription of downstream target genes

The canonical signaling pathway is one of the most extensively studied signaling pathways (Fig. 1). This pathway can be divided into OFF and ON states, during which different biological events occur. Briefly, after WNT proteins are secreted, they are acylated and released into the cytoplasm, where they bind to the receptor FZD and the co-receptors low-density lipoprotein receptor-related proteins 5 and 6 (LRP5/6). In the OFF state, several proteins form the destruction complex with β-catenin, mediating its subsequent ubiquitination and inactivation. In the ON state, the activation of Dishevelled (DVL) inhibits the formation of the destruction complex, allowing β-catenin to escape inactivation and enter the nucleus. Within the nucleus, β-catenin acts as a transcription factor or other roles, promoting the expression of downstream genes. Next, we will detail the mechanism of action of the canonical WNT pathway by examining its major components involved in the biological events, the mechanisms of dysregulation, and their links to cancer.

#### WNT

In canonical WNT signaling pathways, WNT proteins act as ligands that bind to cell membrane receptors, triggering intracellular cascade reactions which regulate diverse cellular processes including proliferation, division, stem cell maintenance, and various stages of embryonic development [5]. The WNT family comprises 19 highly hydrophobic secretory glycoproteins, each characterized by their cysteine-rich domains [6]. The canonical signaling pathway is primarily activated by WNT1, WNT2, WNT3, WNT3A, WNT8B, WNT10A, and WNT10B. In the endoplasmic reticulum, WNTs undergo PORCN-mediated palmitoylation, a modification that is indispensable for membrane receptor binding. The p24 protein family subsequently facilitates the transport of lipid-modified WNTs to the Golgi apparatus for further processing. Mature WNTs are ultimately secreted into the extracellular matrix via exosomes. However, WNT proteins do not represent the exclusive ligands for pathway activation [7]. Norrin binding to FZD4 has been demonstrated as an alternative activation mechanism for WNT signaling [8]. Overexpression of WNTs can mediate cancer phenotypes. For example, transgenic mice with mammary-specific overexpression of WNT1, driven by the mouse mammary tumor virus promoter, develop mammary tumors and serve as a model for breast cancer (BC) research [9]. Non-coding RNAs can bind to the mRNA of WNTs, thereby inhibiting WNT expression and subsequently suppressing the canonical pathway [10]. In addition to overexpression, structural alterations in WNTs proteins, which can be induced by missense mutations of genes, have also been reported. Research has shown that missense mutations in WNTs play an important role in cancer process. For *WNT1*, the Catalogue Of Somatic Mutations In Cancer database contained a total of 83 mutations, most of which were missense mutations [11]. In hepatocellular carcinoma, WNT3 stability and signaling depend on two N-glycosylation sites (asparagine (Asn) 90 and Asn301). Mutation at Asn301 reduces WNT3 stability, while dual mutations at both sites impair its binding to FZD7, downregulate WNT/β-catenin signaling, and attenuate cancer invasion and proliferation [12].

#### FZD

FZD, belonging to the G protein-coupled receptor family, is composed of ten highly conserved seven-transmembrane receptors. FZD and co-receptor LRP bind to WNTs to form trimeric complexes, ultimately leading to intracellular β-catenin accumulation [13]. FZD is thought to be a key receptor molecule in the WNT signaling pathway, and their expression levels influence a series of cellular phenotypes, closely associated with cancer development and progression. Abnormal FZD expression can arise from genetic mutations, mRNA alterations, and dysregulated post-translational modifications. Gene amplification, missense mutations, and truncating mutations are major genetic mechanisms driving cancer through FZD dysregulation. In pancreatic cancer (PCA), gene amplification represents the most prevalent form of FZD mutation. Specifically, *FZD3* and *FZD9* demonstrate amplification mutations [14]. FZD expression levels are also regulated by mRNA state. For example, METTL3-mediated N6-methyladenosine modification of FZD10 mRNA enhances its stability, promoting FZD10 overexpression and hepatocellular carcinogenesis [15]. Non-coding RNAs can downregulate FZD expression by binding to specific mRNA regions; miR-328-3p, for instance, suppresses cancer phenotypes by targeting the 3′-untranslated region of FZD7 mRNA [16]. Post-translationally, FZD levels are negatively regulated by RNF43 and ZNRF3-mediated ubiquitination and degradation. Inactivating mutations in RNF43, particularly in ubiquitination functional domains such as the RING domain or phosphorylation switch, impair its ability to degrade FZD, leading to increased their abundance [17]. Similarly, ZNRF3 mutations elevate FZD levels, although these two ubiquitin ligases target distinct FZD subtypes: RNF43 preferentially regulates FZD1/FZD5/FZD7, while ZNRF3 primarily modulates FZD6 [18]. In addition, the downstream signaling products mediated by FZD can also affect its levels, thereby forming a positive feedback loop. Glenn E. Simmons Jr. et al. demonstrated that β-catenin and c-JUN bind to a region 7 kb upstream of the *FZD7* transcription start site, promoting its expression.

#### LRP5/6

The coreceptors LRP5/6, belonging to LRP superfamily, can form a trimeric complex with FZD and WNT to regulate the activation of WNT signaling, thereby modulating diverse physiological functions. However, dysregulation of LRP5/6 can promote cancer initiation and progression. For instance, Cui, B. et al. discovered that butyrate can suppress properties of BC stem cell. As a histone deacetylase inhibitor, butyrate upregulates ZFP36 expression, which bind to the AU-rich region in the 3'UTR of LRP5 mRNA, thereby inhibiting LRP5 expression and consequently suppressing B stem cell traits [19]. Rong, Z. et al. also found that salt-inducible kinase 2, upon interacting with CK1α, phosphorylates LRP6 at Ser 1490, thereby promoting IDH1 expression via the WNT/β-catenin pathway [20]. In gastric cancer (GC) cells, LRP5 expression is aberrantly elevated and promotes cancer cell proliferation and metastasis by activating the WNT/β-catenin signaling pathway. High LRP5 expression is closely associated with poor prognosis in GC [21]. Epithelial-mesenchymal transition (EMT) is a critical process enabling cancer cell metastasis and invasion. In triple-negative breast cancer (TNBC), overexpressed LRP6 promotes the EMT process in cancer cells by activating the WNT/β-catenin signaling pathway, whereas inhibition of LRP6 using the MESD protein effectively suppresses cell migration and invasion. Furthermore, Ma, J. et al. also identified S100A4 as a downstream target gene of the WNT/β-catenin signaling pathway, which is strongly implicated in tumor metastasis [22]. Similarly, overexpression of LRP6 has been demonstrated to enhance the metastasis and invasion of hepatocellular carcinoma (HCC) cells [23]. In cancer chemotherapy, LRP5/6 is also implicated in cancer cell drug resistance. FOLFOX (5-FU + oxaliplatin (OXA) + leucovorin) induces the expression of alternatively spliced isoforms of CD44 containing variable exon 6 (CD44v6) in colorectal cancer (CRC), which serves as a marker for colorectal cancer-initiating cells. CD44v6 forms a complex with LRP6, promotes its phosphorylation, and consequently augments WNT3A/β-catenin signaling and facilitates drug efflux [24].

#### Destruction complex

When the WNT signaling pathway is inactive due to the absence of WNT ligands, β-catenin, the central component of this pathway, undergoes ubiquitination and proteasomal degradation, maintaining its low cytoplasmic concentration. This process is primarily mediated by the formation of the destruction complex. The widely accepted molecular mechanism involves that casein kinase 1 (CK1) mediates the phosphorylation of Ser45 at the N-terminal domain of β-catenin, followed by the phosphorylation at Thr41, Ser33, and Ser37 residues by glycogen synthase kinase-3β (GSK-3β) [25]. These phosphorylation events facilitate the recruitment of a series of proteins comprising adenomatous polyposis coli (APC), AXIN, GSK-3β, CK1, protein phosphatase 2 A (PP2A), and Wilms tumor gene on the X chromosome (WTX), which orchestrates β-catenin degradation in the cytoplasm. The binding of these proteins to β-catenin marks the formation of the destruction complex. The phosphorylation at Ser33 and Ser37 serves as a recognition signal for β-transducin repeat-containing protein (β-TrCP), enabling subsequent ubiquitination by the Skp1, Cullin1 and β-TrCP ubiquitin ligase complex and proteasomal degradation [26]. In addition, SIAH-1 can also mediate the proteasomal degradation of β-catenin with APC [27].

Aberration of the destruction complex affects the aforementioned steady-state regulation, thereby triggering cancer phenotypes. Next, we will focus on the dysfunction of APC, AXIN, GSK-3β, and CK1, since they are the typical components of the destruction complex. Apc abnormalities represent the most common cause of excessive WNT signaling activation. Truncating mutations in *APC* are particularly prevalent. *APC* mutations are implicated in familial adenomatous polyposis and subsequent CRC, with additional reports of its involvement in HCC and ovarian cancer (OC) [28, 29]. Mechanistically, *APC* truncations disrupt partial binding sites for β-catenin and AXIN, reducing both the assembly efficiency of the destruction complex and its binding capacity to β-catenin. Notably, there are also extensive evidences confirming *APC* mutation-mediated carcinogenesis through WNT-independent pathways [30].

AXIN is divided into AXIN1 and AXIN2, which acts as a scaffold in the formation of the destruction complex. AXIN2 is believed to have tumor-suppressive effects, while the tumor-suppressive role of AXIN1 remains controversial. Skeptics highlight the rare observation of significant β-catenin accumulation in *AXIN1*-mutant HCC, a phenomenon later attributed to compensatory AXIN2 activity mitigating β-catenin elevation [31]. Zhang Ruyi et al. resolved this debate by engineering AXIN1-mutant organoids and employing CRISPR/Cas9-mediated correction, demonstrating that *AXIN1* mutations, which destroy the structure of AXIN1, indeed elevate β-catenin levels [32]. Further studies by Zhang’s group identified three functional domains in AXIN1 regulating WNT signaling in cancer, with deletions in GSK-3β or β-catenin-binding domains critically disrupting β-catenin regulation [30]. Collectively, these findings establish *AXIN1* mutations as β-catenin-dependent drivers of tumorigenesis.

GSK-3β, a core destruction complex component, was historically classified as a tumor suppressor. However, emerging evidence paradoxically links GSK-3β to pro-proliferative effects [33]. Its tumor-promoting activity appears WNT-independent and is not discussed here [34]. Genetic mutations of GSK-3β are rare in cancers, with most mutations localizing to the kinase domain and substrate-binding pocket [35]. These likely impair phosphorylation capacity, potentially enabling WNT pathway activation. Additionally, post-translational modifications (e.g., aberrant phosphorylation) may inactivate GSK-3β to promote malignancy [36].

CK1 exhibits context-dependent roles in cancer, varying by isoform. CK1α acts as a tumor suppressor, while the oncogenic potential of CK1δ/ε remains controversial. CK1 dysregulation in cancer primarily involves alterations in expression levels rather than structural mutations [37]. Reduced CK1 expression, often mediated by promoter methylation, stabilizes β-catenin to drive tumorigenesis [38].

#### DVL

DVL is a key cytoplasmic phosphoprotein that mediates signaling in the WNT pathway. Its domains (DIX, PDZ, DEP) enable interactions with multiple partners, making it essential for embryonic development and tissue homeostasis. DVL is a typical positive regulator of the canonical WNT pathway and plays a role in aberrant WNT signal transduction. Specifically, upon activation of the WNT signaling pathway, FZD recruit and activate DVL [39]. With the assistance of the LRP6 phosphorylation events mediated by GSK-3β and CK1, AXIN is recruited to the plasma membrane and binds to the activated DVL [40]. The disruption of the destruction complex attenuates β-catenin ubiquitination and proteasomal degradation, resulting in cytoplasmic accumulation of β-catenin, which leads to the expression of downstream molecules [41]. In addition to its role at the plasma membrane, DVL can also function within the nucleus, where it interacts with phosphorylated c-JUN and β-catenin to form a DVL–c-JUN–β-catenin–TCF transcriptional complex. For this subcellular localization-dependent regulation, post-translational modifications play indispensable roles. For example, acetylation enhances the nuclear localization of DVL [42]. Another instance is that Foxm1 binds to DVL2 and promotes its nuclear translocation as well as the transcriptional activity of WNT/β-catenin signaling [43]. In addition to playing important roles in aberrant activation of upstream WNT signaling, dysregulation of DVL itself can also drive cancerous phenotypes. Multiple mechanisms may contribute to DVL overexpression. For instance, the loss of non-coding RNAs such as miR-1247-5p leads to upregulation of DVL1, thereby activating canonical signal transduction [44]. Additionally, DVL is extensively degraded during normal WNT signaling, with various proteins involved in maintaining its low steady-state levels. For example, ASPM interacts with DVL2 and inhibits autophagy-mediated DVL2 degradation by reducing its binding to the lipidated form of microtubule-associated protein 1A/1B light chain 3A. CYP2E1 enhances the interaction between DVL2 and the E3 ubiquitin ligase KLHL12 in cells, promoting DVL2 ubiquitination [45]. The DVL ubiquitination process mediated by these proteins can be reversed, leading to abnormal activation of the WNT signaling pathway [46].

#### β-catenin

Β-catenin is a multifunctional protein, and its nuclear accumulation represents the central event in canonical WNT signaling transduction. As mentioned above, in the OFF state of WNT signaling, β-catenin undergoes ubiquitination to prevent its accumulation, whereas in the activated state, the stabilized β-catenin translocates to the nucleus. In the nucleus, β-catenin displaces GRG corepressors and binds to TCF/LEF transcription factors, thereby activating target genes including *c-MYC*, *c-JUN*, *AXIN2* [47, 48]. In addition to the activation of upstream WNT signaling leading to increased nuclear translocation of β-catenin, other biological events can also mediate its nuclear accumulation: (1) Interactions of β-catenin with other non-WNT pathway components proteins. In epithelial cells, the linkage between β-catenin and E-cadherin forms the classic structure mediating cell adhesion [49]. Reduced E-cadherin promote the release of β-catenin, and increased nuclear translocation of β-catenin can further mediate the downregulation of E-cadherin [50]. Additionally, other binding proteins regulate β-catenin homeostasis. For example, FAT, a canonical WNT pathway inhibitor in cervical cancer (CC), acts like a “nail” to anchor β-catenin to the cell membrane, preventing its nuclear entry. Thus, reduced FAT levels lead to β-catenin dissociation and nuclear translocation [51]. Beyond ubiquitinases within the WNT pathway components, other proteins can also mediate β-catenin ubiquitination. SORBS3-β can recruit UBA1 to enhance the degradation of β-catenin, thereby inhibiting cancer cell invasion and migration. Suppression of SORBS3-β contributes to the accumulation of β-catenin, promoting invasive phenotypes [52]. (2) Pre-translational modifications driving abnormal β-catenin expression. β-catenin is produced after transcription and translation of *CTNNB1*. Multiple non-coding RNAs regulate *CTNNB1* expression. For instance, miR-129–2-3p binds *CTNNB1* to suppress its transcription, while LINC01006 acts as a sponge for miR-129–2-3p, upregulating *CTNNB1* expression [53]. CPEB4 interacts with *CTNNB1* mRNA to promote β-catenin expression, facilitating EMT [54]. (3) *CTNNB1* mutations mediating aberrant β-catenin nuclear translocation. *CTNNB1* mutations are a major cause of WNT pathway hyperactivation and have been detected in numerous cancers [55–57]. Mutations in *CTNNB1* are highly concentrated in N-terminal residues, which form the functional domain for β-TrCP, CK-1, and GSK-3β binding. These mutations prevent β-catenin phosphorylation and ubiquitination, enabling escape from degradation and promoting nuclear translocation, ultimately driving oncogenic phenotypes [58]. Notably, certain *CTNNB1*-mutant cancers exhibit distinct histopathological features, such as *CTNNB1*-mutant HCC and trichoblastoma-like high-grade endometrial carcinoma, which may guide precise targeted therapies in clinical practice [59, 60].

### The non-canonical signaling pathways in cancer

Non-canonical signaling pathways are β-catenin-independent signaling pathways including the WNT-Ca^2^⁺ and WNT-planar cell polarity (PCP) signaling pathways. Although the non-canonical and canonical signaling pathways are two entirely distinct branches, they exhibit complex interactions with each other. Experimental evidence demonstrates that both WNT-Ca^2+^ and WNT-PCP pathways antagonize β-catenin-dependent transcription through calcium calmodulin mediated kinase II (CaMKII)- and nuclear factor of activated T cells (NFAT)-mediated transcriptional regulation [61]. Similarly, canonical signaling exerts negative regulation on non-canonical pathways. LRP6, a co-receptor of the canonical signaling pathway, can bind to WNT5A, which preferentially activates the non-canonical signaling pathway, acting as a decoy receptor [62]. Intriguingly, WNT3A, a prototypical canonical ligand, has been shown to activate non-canonical signaling under specific cellular contexts [63]. Given the complex interplay of signaling, it is challenging to study the roles of β-catenin-dependent and β-catenin-independent signaling pathways in cancer independently, although numerous studies have demonstrated the roles of both pathways in promoting and suppressing cancer. Compared with canonical signaling, the molecular mechanisms governing downstream non-canonical signal transduction are still not fully understood. Next, we will describe the currently known molecular mechanisms of the signaling pathways.

The WNT-PCP signaling pathway plays a role in regulating planar cell polarity, manifested by the asymmetric enrichment of proximal and distal transmembrane complexes. Similar to canonical signaling pathways, specific WNTs and FZDs are involved in the activation of the signaling pathway, such as WNT4, WNT5A, WNT5B, WNT7B, and WNT11, as well as FZD3, FZD6, and FZD7 [64]. At the proximal end, the transmembrane complex is primarily composed of Vang-like (VANGL), cadherin EGF LAG seven-pass G-type receptor (CELSR), PRICKLE, inturned (INTU), and DVL. PRICKLE can bind to DVL to prevent the localization of Inversin (INVS) to the proximal end. At the distal end, the transmembrane complex mainly consists of FZD, CELSR, INVS, and DVL. Although the components of the transmembrane complex have been identified, the specific mechanisms of by which WNTs bind to CELSR or VANGL remain unclear [65]. Nevertheless, numerous studies have confirmed the role of VANGL and CELSR dysregulation in cancer progression. Studies have reported that overexpression of VANGL1 and VANGL2 enhances the migratory capacity of cancer cells. In addition to VANGL overexpression, the downregulation of inhibitory factors that bind to VANGL can also lead to hyperactivation of non-canonical WNT signaling. For example, loss of Nrdp1 disrupts its interaction with VANGL, thereby inhibiting DVL ubiquitination and upregulating WNT signaling [66]. Interestingly, Anastas et al. identified a complex containing VANGL1, NOS1AP, and Scribble localized at the leading edge of migrating BC cells. Disruption of this complex reduces tumor cell migration. This complex is not expressed in normal tissues. Thus, VANGL1-mediated formation of this ternary complex may signify enhanced migratory capacity in cancer cells [67]. For CELSR, its expression is significantly higher in lung adenocarcinoma tissues than in adjacent normal tissues, associated with high cancer migratory capacity [68].

**Fig. 2:**
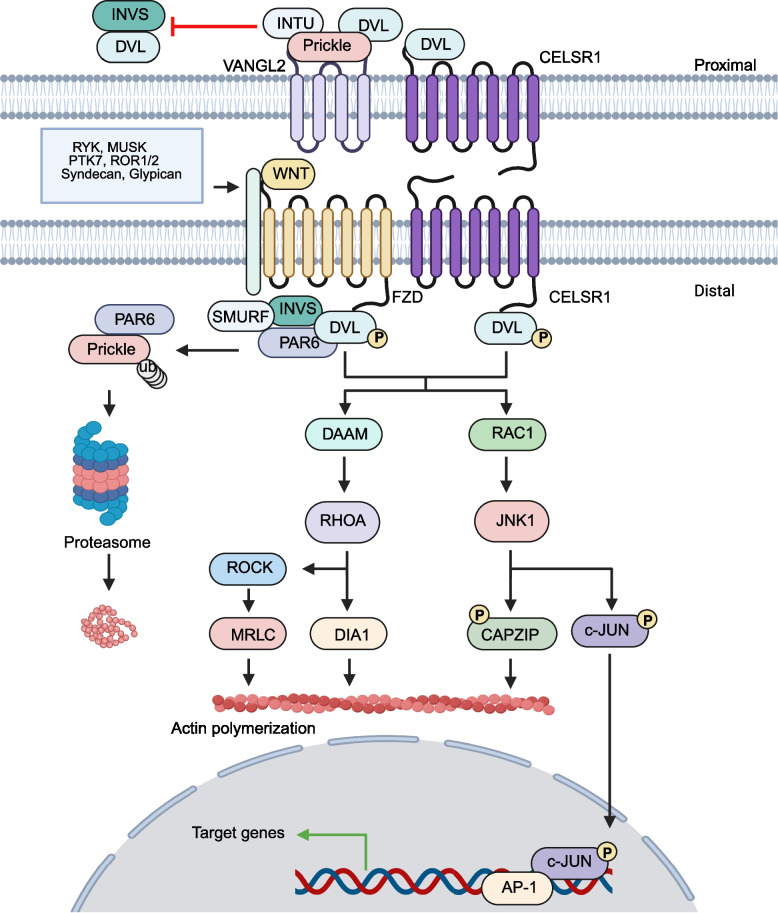
Signal transduction of the WNT-PCP pathway. The core signaling components of the WNT-PCP, such as VANGL and FZD form asymmetrically distributed transmembrane protein complexes on the cell membrane, regulating cell polarity through spatial arrangement. This process is characterized by FZD binding to DVL to initiate downstream signal transduction, while VANGL remains unbound to DVL. Upon activation by FZD, DVL promotes Daam1-mediated activation of RHOA, which subsequently triggers ROCK activation. Concurrently, DVL activates RAC1, thereby facilitating JNK1-dependent phosphorylation of CAPZIP and c-JUN. The activation of the WNT-PCP pathway orchestrates a cascade of downstream events, including actin phosphorylation and transcriptional regulation of target genes

When the WNT-PCP signaling pathway is activated, INVS is localized at the distal end. DVL is found to be phosphorylated, leading to the formation of the DVL-PAR6 complex and the recruitment of SMURF. Subsequently, PRICKLE bound to PAR6 is ubiquitinated and degraded at the distal end [69] (Fig. 2). Currently, multiple co-receptors have been identified to participate in the activation of the WNT-PCP signaling pathway such as receptor-like tyrosine kinase (RYK), muscle-skeletal receptor Tyr kinase (MUSK), and protein tyrosine kinase 7 (PTK7) [70]. Upon the binding of WNT to FZD, DVL is phosphorylated, subsequently leading to the activation of Dvl-associated activator of morphogenesis (DAAM) and RAC1. On one hand, DAAM activates Ras homolog family member A (RHOA), which in turn activates RHO-associated coiled-coil-containing protein kinase (ROCK) and diaphanous 1 (DIA1), with the latter activating mitogen-activated protein kinase (MRLC) [71]. On the other hand, RAC1 activates JNK, leading to the phosphorylation of c-JUN and CapZ-interacting protein (CapZIP). DIA1, MRLC, and CAPZIP all contribute to actin polymerization. Finally, c-JUN translocates to the nucleus to initiate the transcription of target genes [72] (Fig. 2). Cell polarization and changes in motility are the primary phenotypes associated with the activation of the WNT-PCP pathway [73].

In the aforementioned mechanism, RAC1 and RHOA, members of the small GTPase family, determine the mutually antagonistic nature of the two branches of the PCP pathway. RAC1 and RHOA act as molecular switches by cycling between GTP-bound active and GDP-bound inactive states, thereby spatiotemporally regulating cytoskeletal dynamics. Dysregulation of the two components is frequently associated with cancer phenotypes. Multiple mechanisms can lead to aberrant activation of RAC1. In addition to enhanced signaling from upstream WNT ligands and FZD overexpression, dysregulation of post-translational modifications is another key contributor to RAC1 hyperactivation and overexpression. Abnormal phosphorylation and ubiquitination of RAC1 disrupt its GTP-binding capacity, thereby inhibiting signal transduction in the non-canonical pathway. Loss-of-function mutations in the tumor suppressor HACE1 result in a sharp reduction in RAC1 ubiquitination, promoting cancer invasion and migration. Furthermore, alternative splicing or mutations in the *RAC1* gene can impede GTP dissociation or enhance GTP-GDP exchange efficiency, leading to persistent WNT pathway activation [74]. Similarly, defective ubiquitination and phosphorylation can cause RHOA hyperactivation. For instance, the inhibition of phosphorylation at the Ser26 site activate RHOA [75]. Unlike *RAC1* mutations, *RHOA* mutations often downregulate WNT signaling output [76]. For example, a glycine-to-valine substitution in RHOA’s nucleotide-binding pocket introduces a bulky side chain, reducing GTP-binding capacity [77]. Upstream signals, such as FZD overexpression, may also suppress RAC1- and RHOA-mediated WNT pathway activity. Research by C. Fukukawa et al. demonstrated that FZD10 overexpression downregulates RHOA activity, disrupting actin cytoskeletal structures, but upregulates DVL/RAC1/JNK pathway activity to promote synovial sarcoma progression [78]. This suggests that excessive activation of upstream signaling can promote cancer progression through bidirectional regulation of these two pathways.

**Fig. 3:**
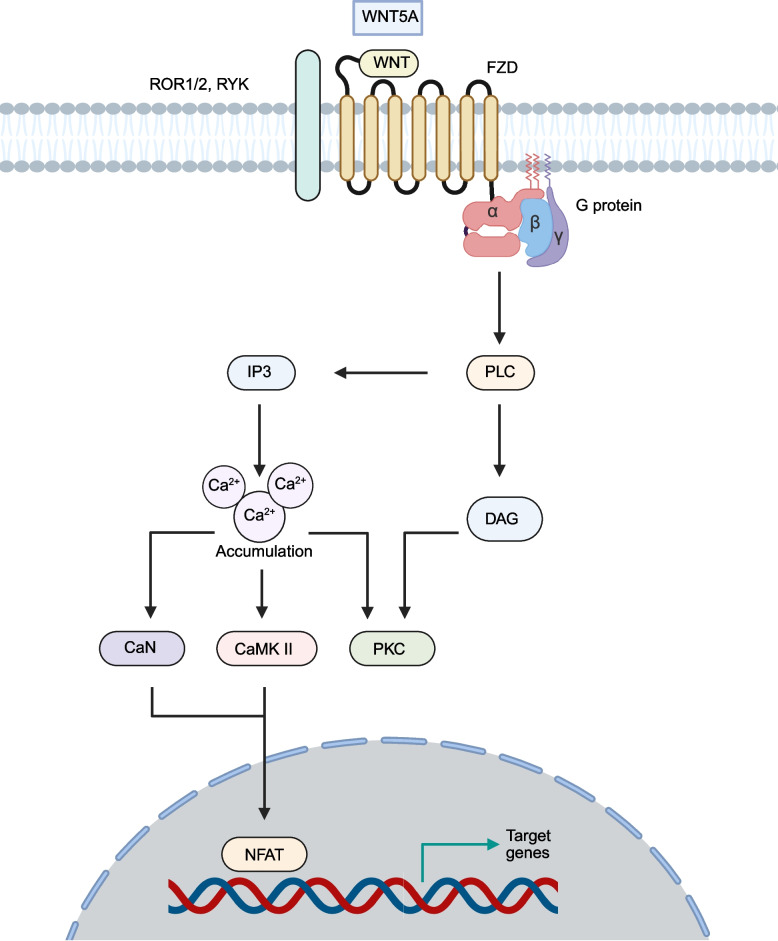
Signal transduction of the WNT-Ca^2+^ pathway. The WNT ligand binds to FZD and its co-receptor, activating heterotrimeric G proteins. The G proteins then mediate the hydrolysis of PLC into IP3, triggering Ca^2+^ release, and into DAG, which activates PKC. The released Ca^2+^ further activate CaN, CaMKII, and PKC, while the generated DAG also contributes to PKC activation. These signaling proteins collectively regulate downstream cellular events. Among them, NFAT serves as a transcription factor, whose activity is modulated by CaN and CaMKII, ultimately driving the transcription of target genes

The WNT-Ca^2^⁺ signaling pathway was first reported in the 1990 s [79]. WNT5A and FZD2 are primarily involved in the activation of the WNT-Ca^2^⁺ signaling pathway [80]. FZD activates coupled heterotrimeric G proteins, which in turn stimulate phospholipase C (PLC). This activation results in the generation of inositol 1,4,5-triphosphate (IP3) and 1,2-diacylglycerol (DAG). IP3 then triggers the release of Ca^2+^ into the cytoplasm, where it accumulates in significant amounts and functions as a second messenger. This Ca^2+^ interacts with CaMKII, calcineurin (CaN), and protein kinase C (PKC) [81]. Subsequently, CaMKII and CaN activate NFAT, leading to the transcription of specific target genes (Fig. 3). During cancer progression, the WNT/Ca^2^⁺ pathway exerts bidirectional regulatory effects, either promoting or suppressing tumorigenesis. Regarding the tumor-suppressive role, WNT5A, a prototypical tumor-suppressive WNT, inhibits cancer progression by activating the WNT/Ca^2^⁺ pathway, thereby enhancing CD8⁺ T-cell cytotoxicity and improving the tumor immune microenvironment [82]. In OC, the WNT/Ca^2^⁺ pathway has been shown to reduce chemotherapy resistance [83]. For the cancer promotion, phosphorylation induces abnormal CaMKII activation, leading to β-catenin phosphorylation and degradation, thereby enhancing cancer cell motility [84]. In prostate cancer (PCa), CaMKII overexpression has been observed. Inhibiting CaMKII reduces PCa cell motility, while CaMKII overexpression also suppresses transcription of β-catenin and its downstream targets [85]. This suggests that the specific role of WNT/Ca^2^⁺ pathway hyperactivation in cancer may depend on its degree of suppression of the canonical WNT pathway. However, the specific switching mechanisms remain to be further elucidated.

Non-canonical pathways play crucial roles in regulating cell polarity, promoting cell motility and invasion, and maintaining stem cell populations [86]. Dysregulation of cellular processes caused by aberration in non-canonical signaling pathways serves as a promoter of tumorigenesis and cancer progression [87]. Therefore, continuous refinement of the molecular framework underlying non-canonical signal transduction is essential for strengthening the established link between aberrant WNT signaling and neoplasia.

### Crosstalk between WNT signaling and other pathways in cancer

**Fig. 4:**
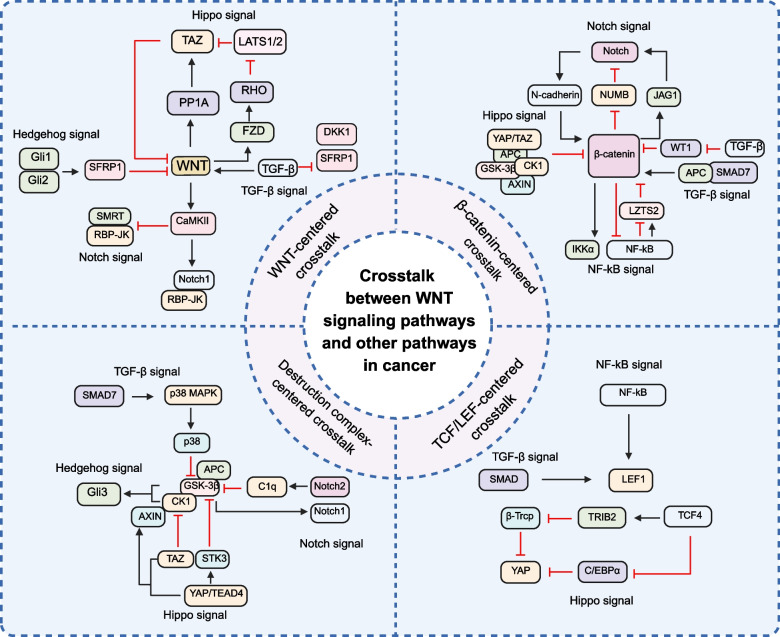
Crosstalk between WNT signaling and other pathways in cancer. The WNT signaling pathway primarily crosstalks with the TGF-β, Hippo, Hedgehog, Notch, and NF-κB signaling pathways, mediated by key components including WNT ligands, β-catenin, the destruction complex, and TCF/LEF transcription factors

Aberrant activation of the WNT signaling pathway is a key driver in the initiation and progression of various cancers. However, within the complex tumor microenvironment, the WNT pathway does not function in isolation; rather, it engages in extensive crosstalk with other signaling pathways, such as the TGF-β, Hippo, Hedgehog, Notch, and NF-κB pathways. This inter-pathway communication is mediated through diverse molecular mechanisms. Here, we map the crosstalk between WNT and other signaling pathways based on the components of the WNT cascade, aiming to provide a comprehensive understanding of the underlying mechanisms (Fig. 4). Such intricate crosstalk enables cancer cells to integrate multiple pro-survival signals, enhancing their proliferative capacity, maintaining stemness, promoting EMT, and conferring resistance to apoptosis. Therefore, elucidating the crosstalk mechanisms between WNT and other pathways is critically important theoretically and clinically, as it may facilitate the development of combination anticancer strategies targeting multiple pathways and help overcome resistance to existing targeted therapies.

#### WNT signaling pathways and TGF-β signaling pathway

The classic transforming growth factor-β (TGF-β) signaling pathway is also known as the SMAD signaling pathway, which acts as the transcription factor for the signaling pathway.TGF-β binds to the Ser/Thr kinase and the tyrosine kinase TβRI and TβRII to form a heteromeric complex, thereby activating the classic TGF-β signaling pathway. Due to conformational changes caused by phosphorylation of TβRI, the GS domain shifts from binding the signaling inhibitor immunophilin FK506 binding protein 1 A (FKBP12) to binding the signaling effectors, the receptor-activated SMADs (R-SMADs). The R-SMADs are activated by phosphorylation modification by TβRI and ultimately enter the nucleus to regulate the transcription of target genes. In addition to the classic TGF-β signaling pathway, TGF-β can also regulate SMAD-independent signaling pathways [88]. TGF-β signaling pathway plays a crucial role in promoting cell proliferation, immune regulation, tissue repair, and extracellular matrix regulation [88]. It has been demonstrated that TGF-β and WNT signaling pathways crosstalk through signaling components such as SMAD, AXIN, DVL, and β-Catenin. TGF-β activates the WNT signaling pathway through multiple mechanisms. For example, TGF-β can inhibit the transcription of DKK1 and SFRP1 in a p38-dependent manner, thereby activating the WNT/β-Catenin signaling pathway [89]. Additionally, TGF-β suppresses the negative regulator Wilms' Tumor 1 of β-catenin, promoting the expression of WNT target genes such as *MMP-9*, *SNAIL1*, *PAI-1*, and *FSP1* [90]. Furthermore, SMAD7 acts as an adaptor protein to recruit p38 MAPK. This recruitment promotes p38 phosphorylation and inhibiting GSK-3β activity by phosphorylating Ser9. Due to GSK-3β inactivation, β-catenin accumulates in the cytoplasm. The C-terminus of SMAD7 binds APC, and its N-terminus binds β-catenin, forming a ternary complex. APC anchors the complex to the plus ends of microtubules, promoting polarized extension and migration of tumor cells [91]. In addition to inactivating GSK-3β to activate β-Catenin, TGF-β can activate the canonical WNT signaling pathway in a β-catenin-independent manner. Experiments have shown that even when LEF1 lacks the β-catenin binding domain (LEF1△20), TGF-β can still activate the WNT signaling pathway through the interaction between SMAD and LEF1 [92]. On the other hand, the WNT signaling pathway can also regulate TGF-β: the WNT signaling pathway mainly exerts its influence on TGF-β by regulating the SMAD linker region. When the WNT signaling pathway is off, GSK-3β phosphorylates the SMAD linker region. This phosphorylation promotes ubiquitination and degradation of SMAD proteins, thereby inhibiting the transcription of target genes of the TGF-β signaling pathway. Additionally, it has been reported that AXIN, through its C-terminal region interacting with SMAD3, can promote the activation of the SMAD3 protein by TβRI, leading to the dissociation of SMAD3 from AXIN and TβRI [93]. Studies have found that blocking one of the signaling pathways affects the activation of the other. For example, the WNT/β-Catenin inhibitor ICG-001 and its derivatives can block TGF-β-induced SMAD2/3 phosphorylation. Deleting TβRII in epithelial cells simultaneously blocks the WNT/β-Catenin signaling pathway and the TGF-β signaling pathway [90].

#### WNT signaling pathways and Hippo signaling pathway

The transcriptional coactivators Yes-associated protein (YAP) and transcriptional coactivator with PDZ-binding motif (TAZ) are characteristic components of the Hippo signaling pathway, whose phosphorylation activities regulate the expression of downstream genes of Hippo [94]. Unphosphorylated YAP/TAZ primarily bind to the TEAD family of transcription factors in the nucleus, activating genes associated with cell proliferation and survival. The Hippo and WNT signaling pathways have been shown to crosstalk through signaling components such as YAP/TAZ, β-Catenin, APC, AXIN, and DVL. YAP/TAZ primarily act as inhibitors of the WNT signaling pathway. In BC, TAZ can disrupt the interaction between CK1 and DVL, thereby inhibiting activation of the WNT3A signaling pathway. Upon knockdown of TAZ, β-Catenin accumulates in the cytoplasm and DVL becomes phosphorylated. YAP/TAZ also directly bind to the N-terminal region of β-catenin through their TEAD-binding domain, preventing β-catenin nuclear translocation and thereby inhibiting WNT target gene expression [95]. However, in GC cells, the Hippo and WNT signaling pathways cooperatively activate each other. The YAP1/TEAD4 complex directly binds to the Serine/threonine kinase 3 (STK3) promoter and promotes its expression, which is a key regulator of the Hippo signaling pathway. STK3 directly binds to the N-terminal domain of GSK-3β, promoting its phosphorylation at Ser9, leading to its inactivation and the cytoplasmic accumulation of β-catenin, thereby promoting malignant phenotypes and 5-FU resistance in GC [96]. Additionally, YAP/TAZ have been shown to be components of the β-catenin degradation complex in the WNT pathway, and the recruitment of β-TrCP depends on YAP/TAZ. Research has demonstrated that when the WNT signaling pathway is inactive, YAP/TAZ directly bind to AXIN1 to become components of the β-catenin destruction complex, which anchors YAP/TAZ in the cytoplasm, inhibiting their nuclear translocation and activation of downstream genes. Concurrently, knockdown of YAP/TAZ significantly reduces the binding of β-TrCP to the AXIN1 complex, which is essential for the ubiquitin-mediated degradation of β-catenin. However, when WNT signaling is activated, AXIN1 binds to the LRP6 receptor, prompting the dissociation of YAP/TAZ from the destruction complex and their entry into the nucleus. In the nucleus, YAP/TAZ, in complex with TEAD transcription factors, cooperate with β-catenin, which itself functions through TCF/LEF transcription factors, collectively driving cell proliferation, maintaining stem cell properties, and promoting tumor growth [97]. The WNT signaling pathway also activates the Hippo signaling pathway. In HCC cells, upon activation of the WNT/β-Catenin pathway, the TCF4 transcription factor directly binds to the enhancer region of the TRIB2 gene, driving its expression. This process relies on FoxA factors to maintain an open chromatin state. TRIB2 acts as an adaptor protein, binding the E3 ubiquitin ligase β-TrCP, thereby blocking its ubiquitin-mediated degradation of YAP and consequently enhancing YAP stability. Furthermore, TRIB2 can downregulate C/EBPα protein levels, relieving the inhibition of YAP transcriptional activity by C/EBPα, thus promoting hepatocyte survival and transformation [98]. Canonical WNT signaling (WNT3A) mediates the dephosphorylation of TAZ at Ser89/306/309 by phosphatase PP1A, inhibiting its binding to 14–3-3 proteins and ubiquitin-mediated degradation, thereby stabilizing TAZ and promoting its nuclear translocation [99]. The β-catenin/TCF complex can bind to a DNA enhancer element within the first intron of the YAP gene, thereby promoting YAP expression. Additionally, a negative feedback mechanism exists between the non-canonical WNT signaling pathway and the Hippo signaling pathway. WNT3A and WNT5A/B promote YAP/TAZ activity through FZD/ROR-Gα₁₂/₁₃-Rho GTPases-Lats1/2, while the activated YAP/TAZ-TEAD complex upregulates the expression of secreted WNT inhibitors, blocking the canonical WNT signaling pathway [100].

#### WNT signaling pathways and Hedgehog signaling pathway

The Hedgehog signaling pathway has two major receptors: the Patched (PTC) and the Smoothened (SMO). When the Hedgehog signal is off, PTC promotes the phosphorylation of CI/GLI by PKA, GSK3, and CK1, followed by the generation of CIR/GLIR-repressed Hedgehog target genes via the Slimb/β-TrCP-mediated ubiquitin and proteasome degradation pathway. Conversely, binding of Hedgehog to PTC enables SMO to transduce signals downstream, altering the balance between the activated form (CIA/GLIA) and the repressed form (CIR/GLIR) of CI/GLI proteins. SMO primarily promotes the formation of CIA/GLIA and thus the expression of target genes by inhibiting the production of CIR/GLIR, stimulating Fu/Ulk3/Stk36-mediated phosphorylation of CI/GLI, and antagonizing Sufu [101]. GLIs serve as a pivotal transcriptional regulator within the Hedgehog signaling pathway. The majority of the crosstalk between the Hedgehog and WNTs signaling pathways revolves around GLIs [102]. For instance, the transcription factors GLI1 and GLI2 directly bind to the *sFRP-1* promoter, modulating its expression. Upon activation of the Hedgehog pathway, a significant increase in sFRP-1 mRNA and protein levels is observed. High expression of sFRP-1 can inhibit WNT1-induced cytoplasmic accumulation of β-catenin [103]. On the other hand, Wang B et al. have elucidated the key molecular mechanism of GLS3 protein processed in the Hedgehog signaling pathway that the C-terminal region of GLI3 is sequentially phosphorylated by PKA, CK1, and GSK3. Subsequently, the WD40 domain of β-TrCP specifically binds to the phosphorylated GLI3 fragment, participating in GLI3 processing. GLI3 undergoes polyubiquitination within the cell, and the proteasome may mediate specific cleavage of GLI3 through partial degradation rather than complete destruction [104]. Additionally, CK1 can also activate GLIs. The crosstalk between Hedgehog and WNT signaling pathways also significantly influences the onset and progression of diseases [105]. In osteoarthritis, Hedgehog induces the expression of a dominant negative isoform of *TCF7L2*, selectively inhibiting β-catenin-induced expression of the fibroblast growth factor 18 gene [106]. In CRC cells, inhibition of the Hedgehog pathway using cyclopamine significantly reduces β-catenin-Tcf transcriptional activity, and this effect can be reversed by exogenous SHH protein, indicating that Hedgehog has a positive regulatory effect on WNT signaling. β-catenin can enhance the transcriptional activity of GLI1, suggesting that WNT signaling may directly regulate the downstream effector of the Hedgehog pathway through β-catenin. Hedgehog-WNT crosstalk drives EMT and tumor invasion cooperatively [107].

#### WNT signaling pathways and Notch signaling pathway

The Notch signaling pathway exhibits intricate crosstalk with the WNT signaling pathway [108]. On one hand, the Notch signaling pathway can regulate the activity of the WNT signaling pathway. In chronic lymphocytic leukemia (CLL), CLL cells express Notch ligands, which bind to Notch receptors on the surface of bone marrow stromal cells (BMSCs), specifically activating Notch2 in BMSCs. This activation is manifested by elevated levels of the Notch2 intracellular domain (ICD) and increased nuclear localization. Notch2 acts as a transcriptional regulator in BMSCs, inducing the expression of complement factor C1q. This, in turn, inhibits GSK3-β activity and reduces its ubiquitin-mediated degradation, thereby stabilizing β-catenin. Additionally, Notch2 upregulates the expression of N-cadherin in CLL cells, further stabilizing β-catenin and enhancing its nuclear translocation and transcriptional activity through interaction with N-cadherin [109]. However, in CRC, ligand of numb protein-X 2, a regulator of Notch, can enhance the activation of the WNT signaling pathway [110]. Aberrant activation of the Notch pathway can also promote the invasiveness and metastasis of oral squamous cell carcinoma (OSCC) by upregulating the expression of c-MYC and β-catenin genes [111]. On the other hand, the WNT signaling pathway primarily facilitates the activation of the Notch signaling pathway. For instance, *Jagged 1* (*JAG1*) is a direct target gene of β-catenin, which can enhance the activity of the Notch signaling pathway by activating the transcription of JAG1 [112]. In BC, keratin 19 (KRT19) binds to the β-catenin/Rac1 complex to inhibit NUMB expression, and relieve NUMB-mediated suppression of the Notch pathway, leading to enhanced Notch signaling [113]. Additionally, KRT19 can directly interact with the β-catenin/RAC1 complex, promoting the nuclear translocation of β-catenin. Enhanced nuclear translocation of β-catenin inhibits the Notch signaling pathway by upregulating NUMB expression. Conversely, KRT19 knockdown suppresses β-catenin nuclear translocation, reduces NUMB expression, and activates the Notch pathway. In CD133/CXCR4/ALDH1 cancer stem cell (CSC), the expression of KRT19 is extremely low, leading to reduced β-catenin nuclear translocation, decreased NUMB expression, excessive activation of the Notch pathway, and consequently enhanced stem cell properties [114]. Gu, B.et al. discovered that the Notch signaling pathway and the WNT/β-catenin signaling pathway exhibit tight cross-regulation during the lineage differentiation of mammary stem cells (MaSCs), and the chromatin effector PYGO2, acting as a histone methylation reader, is the key molecule mediating this regulation by recruiting β-catenin to bind to the Notch3 gene regulatory region, thereby maintaining a chromatin state at this locus characterized by the simultaneous presence of an activating mark (H3K4me3) and a repressive mark (H3K27me3), thus suppressing aberrant Notch3 activation. Loss of PYGO2 causes a shift in the chromatin state towards activation, releasing Notch3 expression. The WNT/β-catenin pathway suppresses Notch3 via PYGO2, maintaining the self-renewal and basal cell characteristics of MaSCs [115]. GSK-3β also mediates the phosphorylation of Ser and Thr on the Notch ICD-1, thereby facilitating its nuclear localization, increasing its transcriptional activity, and enhancing its stability [111]. Beyond the canonical WNT signaling pathway, WNT-Ca^2+^ also interacts with the Notch signaling pathway. WNT5A enhances the transcriptional activity of downstream target genes induced by the Notch1 ICD in a dose-dependent manner, and this process is dependent on CaMKII. Specifically, CaMKII itself can dose-dependently activate the transcription of Notch1 downstream target genes. CaMKII promotes the binding of RBP-Jk to Notch1-IC while inhibiting the binding of RBP-Jk to SMRT by binding to the RID1 domain of SMRT and phosphorylates its Ser-1407 residue, promoting proteasomal degradation of SMRT, thereby enhancing the promoter activity of Notch-responsive genes [116].

#### WNT signaling pathways and NF-κB signaling pathway

Both inhibitor of nuclear factor-κB (NF-κB) subunit α (IKKα) and β (IKKβ) are activators of the NF-κB signaling pathway. However, they exhibit differences in the regulation of β-catenin. IKKβ prevents the nuclear accumulation of β-catenin and inhibits its transcriptional activity, while IKKα promotes its nuclear localization and enhances its transcriptional activity, thereby positively regulating the WNT signaling pathway. The N-terminus of β-catenin contains a conserved sequence DSGXXS, which is the phosphorylation site for IKK [117]. Carayol, N et al. found that IKKα plays an inhibitory role in both the AXIN/APC/GSK-3β complex-dependent phosphorylation and the non-classical SIAH-1-mediated GSK-3β-independent β-catenin degradation pathways. IKKα directly interacts with β-catenin, inhibiting its ubiquitination process, thereby blocking the destruction of β-catenin by the ubiquitin–proteasome system in both degradation pathways [118]. Huang, F.I. et al. also found that in HCC cells and BC cells, there are multiple NF-κB binding sites upstream of the LEF1 transcription start site. NF-κB can directly bind to these sites upstream of the LEF1 transcription start site, thereby activating LEF1 transcription [119]. Furthermore, in MCL cells, β-catenin and NF-κB form a complex through protein–protein interactions, bind to the NF-κB DNA consensus sequence, and directly promote the transcription of NF-κB target genes. Ma, B. et al. discovered that NF-κB regulation of the β-catenin/TCF pathway is cell type-specific: In colon, liver, and BC cells, NF-κB activation can upregulate LZTS2, which binds to β-catenin, preventing its entry into the nucleus and inhibiting its transcriptional activity. However, in glioma cells, NF-κB instead downregulates LZTS2 to promote the activity of β-catenin [120]. Chang, J. et al. found that in mesenchymal stem cells (MSCs), the pro-inflammatory factors TNF and IL-17 can activate the IKK–NF-κB signaling pathway. TNF induces the mRNA expression of SMURF1 and SMURF2 via IKK–NF-κB, while IL-17 induces SMURF2 in an IKK-dependent manner. After TNF treatment, the NF-κB subunit p65 directly binds to the promoter regions of SMURF1 and SMURF2 and promotes their expression, which inhibits the WNT/β-catenin signaling pathway [121]. In addition to its antagonistic role, the Ectodysplasin A (EDA)/EDA receptor/NF-κB pathway also activates the expression of epithelial Shh and Cyclin D1, which are likewise target genes of the WNT signaling pathway [122].The WNT signaling pathway also exerts regulatory effects on NF-κB. In human colon and BC cells, overexpressed β-catenin can form a complex with the RelA and p50 subunits of NF-κB, reducing NF-κB's DNA-binding ability and transcriptional activity, thereby inhibiting the expression of its target genes. However, in endometrial cancer (EC) cells, NF-κB's RelA can compete with β-catenin for binding to the co-activator p300, inhibiting the transcriptional activity of β-catenin/TCF [120]. Researchers have found that WNT/β-catenin can transcriptionally upregulate coding region determinant-binding protein (CRD-BP), which binds and stabilizes the mRNA of β-TrCP, promoting β-TrCP-mediated degradation of IκB-α and activating the NF-κB signaling pathway [123].

## Dysregulation of the WNT pathway in oncogenesis across various cancers

The WNT signaling pathway, as a core regulatory hub in tumor progression, is abnormally activated in driving the advancement of 30%−40% of malignant tumors, including BC, CRC, Glioblastoma multiforme (GBM), HCC, and PCa. External stimuli, such as gut microbiota, can induce this abnormal activation, driving oncogene expression through the β-catenin/TCF transcription complex. This pathway exhibits significant tissue-specific regulatory patterns: in CRC, APC mutations primarily drive pathway activation, while in HCC, *CTNNB1* mutations are more common. Here, we systematically elucidate the mechanistic underpinnings through the lens of both canonical and non-canonical WNT signaling pathways in different cancers.

### Breast cancer

Within the canonical WNT/β-catenin pathway, the aberrant nuclear accumulation of β-catenin represents the central oncogenic event [25]. In BC, this phenomenon is predominantly orchestrated through the dysregulation of three key regulatory nodes: WNT ligands, FZD/LRP receptor complexes, and the β-catenin destruction complex. Sustained activation of WNT ligands not only initiates mammary tumorigenesis but also facilitates metastatic progression [124]. Therapeutic intervention targeting specific FZD receptor subtypes (e.g., FZD1/2/7) effectively attenuates β-catenin nuclear translocation, resulting in significant reduction of tumor-initiating cell populations and suppressed growth of basal-like breast tumor organoids [125]. AXIN, the central scaffolding protein of the destruction complex, undergoes phosphorylation at Ser45, which impairs its function in β-catenin degradation, thereby promoting β-catenin stabilization and nuclear accumulation [126]. β-catenin nuclear translocation can occur via mechanisms that bypass canonical WNT upstream signaling. Recent studies reveal that SHC4 mediates phosphorylation of Src kinase at tyrosine 419, which in turn catalyzes phosphorylation of β-catenin at critical residues that facilitate nuclear translocation. This signaling cascade potently upregulates key mediators of BC invasion and metastasis, including CD44 and MMP-7 [127]. Moreover, the RNA helicase DEAD-box helicase 5 (DDX5) functions as a molecular chaperone that protects β-catenin from proteasomal degradation, while Aurora-A kinase interacting protein 1 (AURKAIP1) enhances this stabilization by preventing ubiquitin-mediated degradation of DDX5. Together, these interactions create a positive feedback loop that amplifies β-catenin signaling and accelerates BC progression [128].

Non-canonical pathways also play crucial roles in BC progression. The WNT-PCP pathway is associated with the efficacy of various BC therapeutics. Upregulation of the WNT-PCP has been demonstrated to correlate with resistance to anti-estrogen therapy and doxorubicin in BC [129]. This doxorubicin resistance may be indirectly mediated through activation of the WNT5A/ROR2/c-JUN signaling axis. Complementary to cellular and molecular studies, bioinformatics analysis identifies WNT5A/ROR2 as a prototypical WNT-PCP pathway in BC, showing significant co-expression with IL16, SULF1, PLXNA2 and RASGRF2, while predicting responsiveness to MEK1/2 inhibitors [130]. Beyond WNT5A, WNT11 may participate in ROR2-mediated signaling to promote BC invasiveness [131]. The role of WNT-Ca^2+^ signaling in BC development remains controversial. On one hand, WNT-Ca^2+^ signaling suppresses canonical WNT transduction, where elevated cytosolic Ca^2+^ activates CaMKII to promote β-catenin phosphorylation and degradation, consequently reversing EMT [132]. Conversely, calcineurin activation enhances NFATC3 nuclear translocation to stimulate vascular endothelial growth factor expression, angiogenesis and tumor growth [133]. These paradoxical findings underscore the need for more comprehensive investigations into WNT-Ca^2+^ signaling in BC. Elucidation of these mechanisms provides potential therapeutic targets for clinical BC management.

### Colorectal cancer

In contrast to BC, aberrant activation of the WNT signaling pathway in CRC predominantly stems from mutations in WNT pathway component genes. These WNT-related gene mutations show the highest incidence in rectal cancers [134]. Luís Nunes et al. established a comprehensive mutational landscape of CRC, revealing recurrent mutations in WNT pathway genes including *APC*,* AXIN2*, *CTNNB1*, *RNF43*, *TCF12 and TCF7L2* [135]. Another study demonstrated age-dependent mutation patterns, with *RNF43* mutations predominating in early-onset CRC versus *APC* mutations in adult-onset cases [136]. *APC* mutations represent the principal lesions driving WNT hyperactivation in CRC, comprising missense mutations and more prevalent truncating variants [137]. *APC* mutations inactivate the destruction complex, impairing β-catenin degradation and promoting nuclear accumulation [138]. Recent studies have provided mechanistic refinements to this paradigm, demonstrating that AXIN undergoes liquid–liquid phase separation to organize destruction complex condensates under physiological conditions [139]. *APC* truncations disrupt complex assembly by impairing recruitment of GSK-3β and CK1α into these liquid droplets [140]. Truncated APC also fails to suppress USP10, which stabilizes β-catenin through direct binding [141]. HMGA1 amplifies WNT signaling output from *APC* mutations, potentiating tumorigenesis [142]. Given its pivotal role, researchers identified a distinct APC truncation (lacking seven 20-amino acid repeats) as a biomarker for β-catenin inhibitor resistance in CRC therapy [143]. The gut microenvironment enables microbial interactions, exemplified by Clostridium_sensu_stricto_1 correlating with β-catenin, AXIN2 and c-MYC expression [144]. These findings illuminate the multifaceted complexity of canonical WNT dysregulation, particularly through APC mutations, in colorectal carcinogenesis.

There is evidence indicating aberrant activation of non-canonical WNT signaling in CRC. Collagen type I α 1 overexpression enhances c-JUN N-terminal kinase phosphorylation and upregulates RHOA-GTP expression [145]. WNT5A/JNK signaling promotes CSC development with β-catenin activity and modulates tumor microenvironment by regulating M2 macrophage polarization and T-cell differentiation [146]. In colitis-associated CRC, WNT5A activates CaMKII to subsequently stimulate STAT3-mediated tumor progression [147]. These findings demonstrate that both the WNT-PCP and WNT-Ca^2+^ pathways can facilitate CRC progression. However, certain non-canonical pathways exhibit tumor-suppressive effects, as evidenced by Evacetrapib-induced JNK activation inhibiting CRC cell growth [148]. One review article reveal the dual roles of non-canonical signaling in CRC pathogenesis, with tumor suppression mediated through ROR2-dependent signaling, competitive antagonism of canonical pathways, and downstream gene regulation [149]. Intriguingly, patients exhibiting low β-catenin but high TCF12/MALAT1/Cyclin D1 expression demonstrate poorer prognosis, a phenotype strongly associated with non-canonical WNT activation [150]. These observations warrant caution regarding the oncogenic potential of non-canonical WNT signaling in CRC, notwithstanding its duality in modulating cancer progression.

### Glioblastoma multiforme

Similar to BC, GBM rarely harbor mutations in core components of the WNT pathway [151]. The GBM phenotype shows strong correlation with hyperactivated WNT signaling, particularly the WNT/β-catenin axis. Aberrant activation of canonical WNT signaling results from dysregulation at multiple nodal points. Non-coding RNA-mediated pre-translational regulation plays an indispensable role in this process. For instance, circMMD functions as a miR-15b-5p sponge, attenuating miR-FZD6 mRNA interaction to upregulate FZD6 expression. Moreover, circMMD enhances DVL1 expression, dually promoting GBM cell proliferation [152]. RNA-binding proteins like YTHDF2 facilitate β-catenin nuclear translocation by degrading APC and GSK-3β mRNAs, thereby hyperactivating canonical WNT signaling [153]. Enhanced nuclear β-catenin elevates expression of canonical WNT targets including *c-MYC* and *survivin* [154]. Interestingly, c-MYC acts as a transcription factor for *CTNNB1* in GBM cells, establishing a positive feedback loop [155]. Canonical WNT activation promotes multiple oncogenic phenotypes in GBM cells. The WNT/β-catenin axis upregulates *neuroligin 3* transcription to induce CSC formation in neighboring cells [156]; it also enhances chemoresistance [154], EMT progression [157], and invasiveness with colony-forming capacity [158].

Therapeutic targeting of canonical WNT signaling in GBM remains largely ineffective, suggesting alternative pathways may drive tumor aggressiveness. Core WNT-PCP components VANGL1, VANGL2, FZD7 and DAAM1 play important roles in GBM progression. DAAM1 demonstrates the most pronounced dysregulation with concomitant upregulation of downstream effectors RHOA, ROCK2 and RAC2 [159]. VANGL1/2 mediate essential physiological functions including ciliary elongation and neural tube patterning via the WNT-PCP signaling [160]. Dreyer et al. demonstrated VANGL1-FZD7 complex formation at leading edges of migrating GBM cells, driving actin cytoskeletal reorganization [161]. Overexpressed VANGL2 promotes migration, invasion, proliferation and tumor sphere formation, representing a potential prognostic biomarker [162]. Emerging evidence implicates WNT-Ca^2+^ signaling in GBM pathogenesis. Huang et al. revealed the dual regulatory role of WNT-Ca^2+^ signaling in GBM cells. FZD6-mediated CaMKII activation upregulates NLK to antagonize canonical WNT, while parallel STAT3/NF-κB activation sustains tumor growth and renewal [163].

### Hepatocellular carcinoma

HCC, a prevalent malignancy of the digestive gland, frequently exhibits WNT pathway dysregulation primarily driven by *CTNNB1* mutations. *CTNNB1* mutagenesis correlates with multiple etiological factors, particularly alcohol consumption, viral infection, and aflatoxin exposure [164]. Recurrent mutations cluster at exon 3 Ser/Thr residues or adjacent amino acids [165], disrupting phosphorylation sites required for β-catenin ubiquitination and proteasomal degradation, thereby stabilizing nuclear β-catenin. *CTNNB1*-mutant HCC constitutes a distinct subtype characterized by well-differentiation, pseudoglandular patterns, cholestasis, and immune exclusion [166]. Mechanistic investigations have elucidated the molecular basis of this unique HCC subtype. T-box transcription factor 3 mRNA is upregulated in *CTNNB1*-mutant HCC, correlating with enhanced differentiation status [167]; β-catenin overexpression induces ATP-binding cassette transporter expression, driving profound cholestasis [168]; Nuclear β-catenin transactivates PF4, expanding myeloid-derived suppressor cell populations while depleting CD8^+^ T cells, potentially mediating immune evasion [169]. However, comprehensive understanding of *CTNNB1*-driven transcriptional programs and their phenotypic consequences remains incomplete, warranting further investigation. Beyond *CTNNB1* mutations, β-catenin hyperactivation occurs through FZD7/10 overexpression [15, 170], destruction complex phosphorylation [171], and translational dysregulation [172]. This orchestrates oncogenic transcriptional cascades involving YAP-1, c-JUN, CD90, epithelial cell adhesion/activating molecule, CD44, and CD133, enhancing stemness, chemoresistance, and metastatic competence [173].

The role of non-canonical pathways in HCC progression exhibits considerable complexity. The tumor-modulating functions of non-canonical signaling, particularly the WNT-PCP pathway, require further elucidation. The initial 2012 research identified WNT5A/ROR2 downregulation as correlating with poor prognosis [174]. Wakizaka et al. demonstrated that WNT5A/ROR2 signaling promotes HCC differentiation, with high expression predicting favorable outcomes and WNT5A/ROR2-negative cases showing elevated Alpha-Fetoprotein (AFP) levels [175]. Contrastingly, Huang et al. reported that WNT5A/ROR2 activation suppresses canonical signaling, with this pathway switching mechanism driving HCC progression [176]. Given the limited impact of *CTNNB1*/*AXIN* mutations on downstream WNT targets [164], non-canonical pathways may play a more important role in HCC progression, though whether WNT5A/ROR2 serves as the principal mediator remains unresolved. Beyond WNT5A/ROR2, upregulated FZD2/ROR1 signaling similarly enhances EMT in HCC cells [177]. WNT5A activates this axis, potentially through STAT3 phosphorylation to promote oncogenic phenotypes. FZD2, WNT5A and ROR2 collectively represent potential prognostic biomarkers [175, 178].

### Prostate cancer

PCa is one of the most common malignant tumors in men, particularly prevalent among older males. While early-stage PCa typically grows slowly and has a favorable prognosis, advanced or aggressive forms can lead to high mortality rates. Androgen receptor (AR) expression levels determine the efficacy of anti-androgen therapy, a cornerstone treatment for PCa. The WNT signaling pathway modulates AR expression in PCa cells, directly influencing therapeutic resistance. Alterations in canonical WNT components impact AR receptor expression. Beildeck et al. systematically delineated the β-catenin-AR crosstalk. β-catenin potentiates AR transcriptional activity through direct binding, a function mutually exclusive with its canonical TCF/LEF-mediated signaling [179]. This mechanism becomes prominently activated during castration-resistant progression [180]. Beyond AR upregulation, canonical WNT signaling drives metastatic progression through multiple avenues. β-catenin stabilization, FZD/WNT activation, or destruction complex impairment all promote metastasis via WNT hyperactivation [181–183], though definitive pro-metastatic target genes remain unvalidated. Canonical WNT signaling additionally governs CSC acquisition, EMT progression, and immune microenvironment remodeling [184, 185].

Extensive research has elucidated the multifaceted mechanisms of non-canonical WNT signaling in PCa, which can be categorized as follows. (1) FYN/STAT3 pathway: Representing a distinct non-canonical axis independent of WNT-PCP/Ca^2+^ signaling, this pathway is activated by FZD2/8 receptors. The scaffolding protein Abi functions as a molecular gatekeeper between FZD2 and FYN/STAT3, critically regulating EMT progression [186, 187]. (2) The WNT-PCP pathway: Core components including ROR1/ROR2 demonstrate overexpression, with the kyoto encyclopedia of genes and genomes (KEGG) analysis revealing differential expression of DAAM1/RAC effectors [188, 189]. However, direct experimental validation of complete WNT-PCP cascades and their transcriptional targets remains lacking. (3) The WNT-Ca^2+^ pathway: WNT5A overexpression induces calcium oscillations that activate CaMKII, enhancing wound healing and motility in malignant cells [85]. KEGG data further implicates PLC and calcineurin dysregulation, suggesting concurrent activation of multiple Ca^2+^-dependent mechanisms [188].

### Other relevant cancers

The WNT pathway plays a significant role in various other cancers. Targeting upregulated β-catenin in Renal cell carcinoma (RCC) is considered a promising strategy. Alterations in multiple WNT pathway components, such as WNT9B, FZD2/7, AXIN, and APC, can lead to the upregulation of β-catenin [190–192]. CLIP170, a protein implicated in multiple cancers, promotes the nuclear translocation of β-catenin by directly mediating the reduction of its ubiquitination levels, thereby increasing FOSL1 expression and facilitating the proliferation and growth of RCC cells [193]. Similar to RCC, the canonical WNT pathway also plays a crucial role in melanoma. A recent study demonstrated that WNT3A binds to FZD and LRP6 to promote the nuclear translocation of β-catenin, subsequently enhancing the expression of various oncogenes, including *c-MYC, MMP-9, SOX10, and CD44* [194]. Regarding the progression of melanoma, Pan Song et al. proposed that melanoma employs distinct WNT signaling pathways during its growth [151]. The canonical WNT pathway primarily promotes growth and transformation to enhance proliferative capacity [195], whereas non-canonical WNT signaling appears to facilitate melanoma metastasis and invasion [196]. Additionally, upregulation of WNT5A may enhance the immunogenicity of melanoma [197], though this conclusion remains controversial. For instance, FZD6 upregulation promotes melanoma invasion via the canonical WNT pathway without affecting proliferation, while FZD3 enhances melanoma growth and metastasis independently of the canonical WNT pathway [198]. Thus, the specific roles of different WNT pathways may still be closely associated with the types of FZD receptors involved [199]. Osteosarcoma (OS) is one of the most metastatic cancers, and the WNT pathway is highly active in its progression. The canonical WNT signaling not only plays a critical role in tumor invasion and EMT but is also closely linked to angiogenesis, cell proliferation, and drug resistance in OS [200]. However, Singla A. et al. suggested that the role of canonical WNT signaling in OS is less defined compared to other cancers [201]. Evidence indicates that DKK-1, an inhibitor of canonical WNT signaling, may paradoxically promote tumor phenotypes, likely due to its low specificity, which enhances other signaling pathways and counteracts the inhibitory effects of canonical WNT signaling [202]. Moreover, since canonical WNT signaling promotes the differentiation of MSCs into osteoblasts in normal human tissues, its inhibition may lead to the acquisition of an MSC-like phenotype, potentially explaining why some researchers consider canonical WNT signaling suppressive in OS progression [203]. Non-canonical WNT signaling also contributes to OS, with evidence suggesting its role in promoting ALDH-1 expression to facilitate cancer phenotypes [203]. The WNT pathway is essential for the development of oral and associated organs [204], and its dysregulation can contribute to oral carcinogenesis. Both canonical and non-canonical pathways play significant roles in oral cancer progression, including metabolic reprogramming, acquisition of cancer stemness, immune microenvironment modulation, and EMT [205–208]. In lung cancer (LCA), WNT signaling is also pivotal. The canonical WNT pathway promotes various tumor phenotypes in non-small cell lung cancer (NSCLC), such as proliferation, migration, and drug resistance [209, 210]. Additionally, multiple non-canonical WNT components have been identified in LCA [211–213], but similar to other cancers, their pro- or anti-tumor effects require further clarification. Furthermore, WNT signaling plays a critical role in the pulmonary metastasis of other cancer types [177, 214, 215].

## Potential therapeutic approaches targeting WNT signaling

**Fig. 5:**
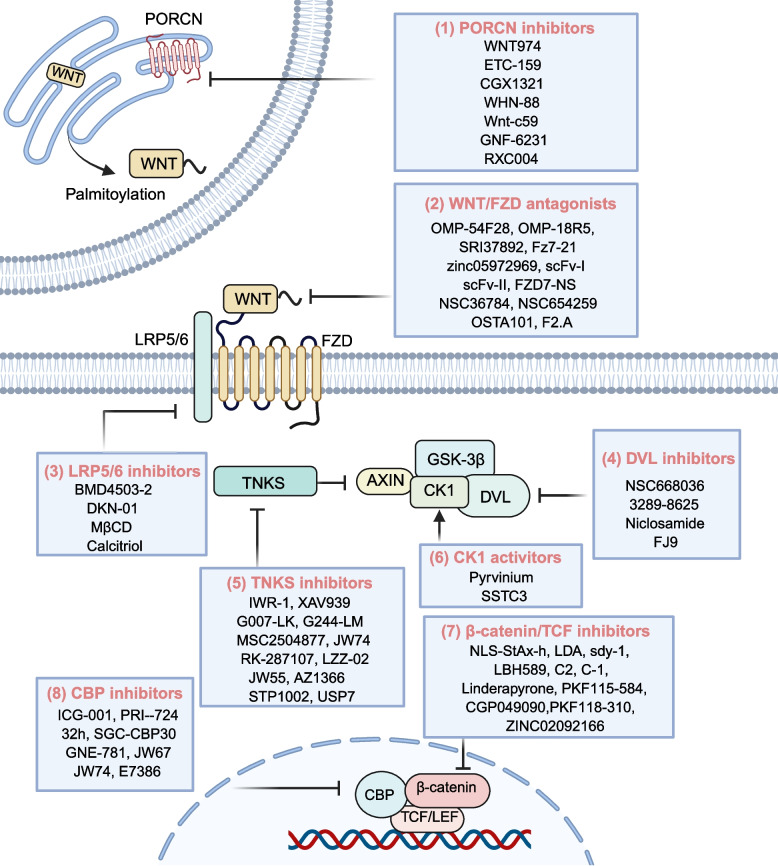
WNT signaling pathway inhibitors. The WNT signaling pathway is a promising target for cancer therapy. Currently, numerous inhibitors have been reported in both preclinical and clinical trial stages. Based on the targeted components of the WNT pathway, these inhibitors can be categorized into the following classes: (1) Porcupine inhibitors, (2) WNT/FZD antagonists, (3) LRP5/6 inhibitors, (4) DVL inhibitors, (5) TNKS inhibitors, (6) CK1 agonists, and (7) β-catenin/TCF transcriptional complex inhibitors, (8) CBP inhibitors

**Fig. 6:**
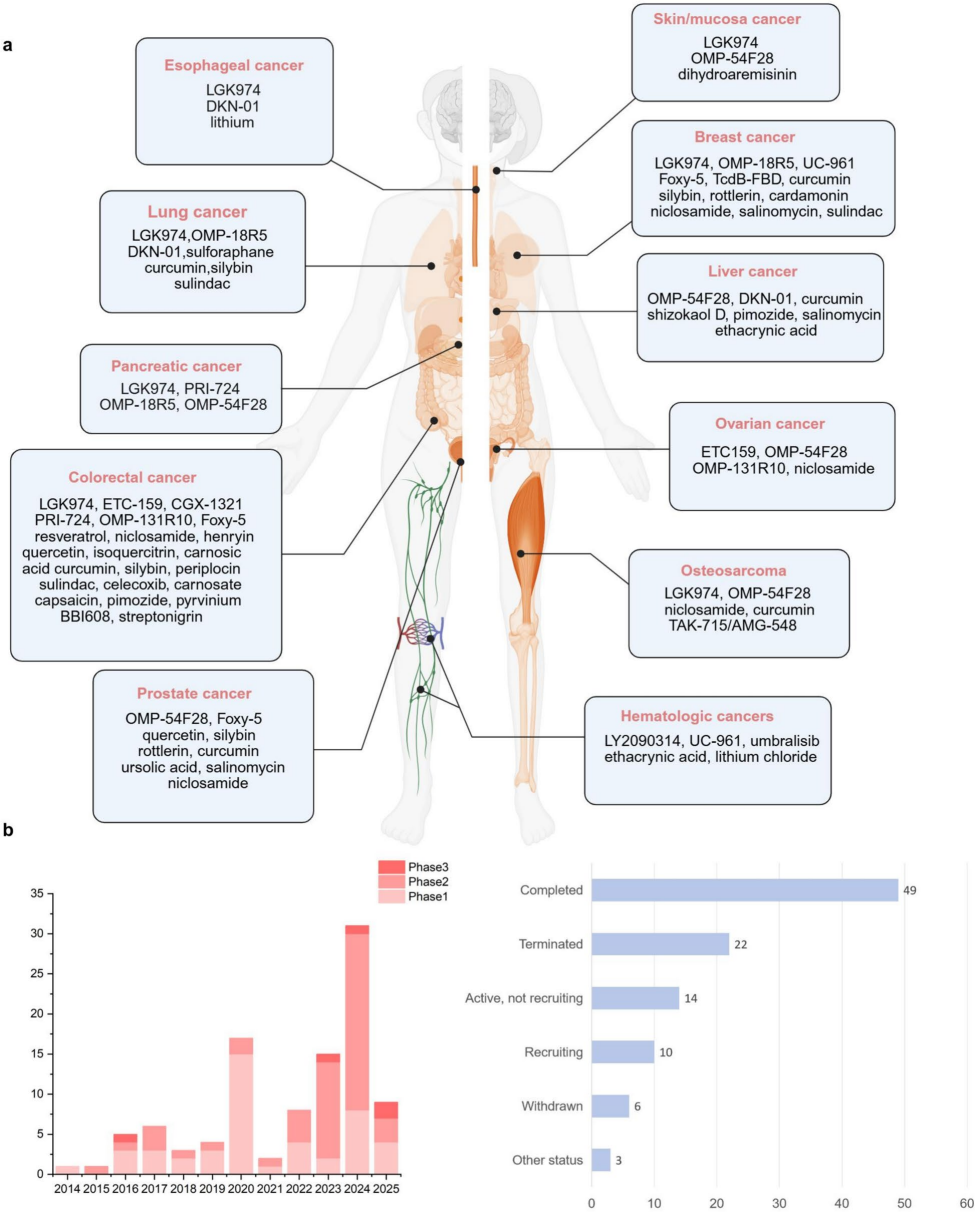
Comprehensive landscape of clinical trials about WNT pathway inhibitors (data cutoff: March 2025). **a** The summary of the WNT signaling inhibitors currently registered in clinical trials. WNT signaling inhibitors targeting the same type of cancer are grouped within a single diagram. **b** Statistics on clinical trials of currently available WNT signaling pathway inhibitors. Annual distribution of clinical trials, with color coding indicating trial phases: light red for Phase 1, medium red for Phase 2, and dark for Phase 3. Current status of clinical trials, classified into five distinct categories: “Completed” “Terminated” “Active, not recruiting” “Withdrawn” “Other status”

The aberrant activation of the WNT signaling pathway has been confirmed to be closely associated with the initiation and progression of various malignancies. This discovery has positioned targeting critical regulatory nodes within this pathway as a highly promising anticancer strategy. Research indicates that dysregulation of this pathway not only contributes to malignant tumor transformation but is also implicated in the pathological processes of multiple degenerative diseases. Currently, inhibitors targeting distinct components of the WNT/β-catenin signaling cascade have shown significant progress in development, with several drug candidates advancing into preclinical evaluations (Fig. 5) or various phases of clinical trials (Table 1 and Fig. 6). These novel therapeutic agents effectively suppress canonical WNT signal transduction through mechanisms such as selectively interfering with WNT ligand binding, disrupting β-catenin stability, or blocking the formation of transcriptional complexes, thereby achieving targeted regulation of tumor growth.

**Table 1:** The Summary of Clinical Trials Targeting the WNT Signaling Pathway at Different Stages

Drug	NCT	Phase	Status	Last Update Posted	Cancer Type
WNT947	NCT02649530	Phase 2	Withdrawn	2016/9/30	SCC
WNT947	NCT02278133	Phase 1/2	Completed	2017/10/9	MCRC
WNT947	NCT01351103	Phase 1	Completed	2024/9/19	PCA BRAF Mutant CRC Melanoma
NUC-7738	NCT03829254	Phase 1/2	Recruiting	2024/11/12	Lymphoma
DKN-01	NCT03645980	Phase 1/2	Unknown status	2020/10/22	HCC
DKN-01	NCT01457417	Phase 1	Completed	2016/9/28	Multiple Myeloma NSCLC
DKN-01	NCT04166721	Phase 1/2	Active, not recruiting	2024/8/2	Metastatic EC Metastatic GC
DKN-01	NCT02013154	Phase 1	Completed	2023/11/1	Esophageal Neoplasms Adenocarcinoma of the Gastroesophageal Junction Gastroesophageal Cancer (GEC) SCC GA
DKN-01	NCT04363801	Phase 2	Active, not recruiting	2024/1/22	GC GA GEC
DKN-01	NCT01711671	Phase 1	Completed	2017/3/13	MultipEndometrial Cancerle Myeloma
DKN-01	NCT05761951	Phase 2	Recruiting	2024/11/18	
DKN-01	NCT04681248		Available	2023/9/29	Esophageal Neoplasm Adenocarcinoma of the Gastroesophageal Junction|GEC SCC GA EC Uterine Cancer OC Carcinosarcoma GC
DKN-01	NCT02375880	Phase 1	Completed	2018/9/10	Carcinoma of Intrahepatic and Extra-hepatic Biliary System Bile Duct Cancer Carcinoma of Gallbladder Cholangiocarcinoma
DKN-01	NCT03395080	Phase 2	Completed	2023/7/12	EC Uterine Cancer OC Carcinosarcoma
DKN-01	NCT03837353	Phase 1/2	Terminated	2023/12/18	PCa
DKN-01	NCT05480306	Phase 2	Active, not recruiting	2024/9/27	CRC Colorectal Adenocarcinoma Colo-rectal Cancer
DKN-01	NCT03818997	Phase 2	Withdrawn	2020/2/5	EC BTC GEC Hepatobiliary Neoplasm
DKN-01	NCT04057365	Phase 2	Terminated	2024/4/10	BTC
Vantictumab	NCT01957007	Phase 1	Completed	2020/9/9	Solid Tumors
Vantictumab	NCT01973309	Phase 1	Completed	2020/9/9	Metastatic BC
Vantictumab	NCT02005315	Phase 1	Completed	2020/9/9	Stage IV PCA
OMP-54F28	NCT02069145	Phase 1	Completed	2020/8/11	Hepatocellular Cancer HCC
OMP-54F28	NCT02092363	Phase 1	Completed	2020/8/12	OC
OMP-54F28	NCT01608867	Phase 1	Completed	2020/8/11	Solid Tumors
OMP-54F28	NCT02050178	Phase 1	Completed	2020/9/9	Stage IV PCA
RXC004	NCT03447470	Phase 1	Completed	2025/1/29	Cancer Solid Tumor
XNW7201	NCT03901950	Phase 1	Completed	2023/2/16	Advanced Solid Tumors
FOG-001	NCT05919264	Phase 1/2	Recruiting	2024/12/17	CRC
Sinecatechins	NCT02029352	Phase 2/3	Completed	2016/5/18	Carcinoma, Basal Cell
Calcitriol	NCT01358045	Phase 2	Completed	2015/1/13	Basal Cell Carcinoma
Resveratrol	NCT00256334	Phase 1	Completed	2014/6/20	CRC
PTK7-ADC	NCT03243331	Phase 1	Completed	2021/1/7	TNBC Metastatic BC
ETC-159	NCT06513624	Phase 1	Not yet recruiting	2024/11/29	With MSS/pMMR Advanced, Platinum-resistant OC
ETC-159	NCT02521844	Phase 1	Active, not recruiting	2024/10/8	Solid Tumors
CGX1321	NCT03507998	Phase 1		2020/7/24	Colorectal Adenocarcinoma GA Pancreatic Adenocarcinoma
CGX1321	NCT02675946	Phase 1		2022/1/26	Solid Tumors Gastrointestinal Cancer
OMP-18R5	NCT01345201	Phase 1	Completed	2020/9/9	Solid Tumors
OMP-18R5	NCT02005315	Phase 1	Completed	2020/9/9	Stage IV PCA
OMP-18R5	NCT01957007	Phase 1	Completed	2020/9/9	Solid Tumors
OMP-18R5	NCT01973309	Phase 1	Completed	2020/9/9	Metastatic BC
OMP-131R10	NCT02482441	Phase 1	Completed	2020/8/11	Advanced Relapsed Tumors Refractory Solid Tumors
UC-961	NCT02222688	Phase 1	Completed	2020/8/13	CLL
UC-961	NCT05156905	Phase 1	Terminated	2024/10/9	Metastatic Castration-resistant Prostate Cancer
UC-961	NCT02860676	Phase 1	Completed	2019/3/20	CLL
UC-961	NCT04501939	Phase 2	Active, not recruiting	2023/11/28	CLL
UC-961	NCT02776917	Phase 1	Completed	2024/4/10	Breast Neoplasms
UC-961	NCT03088878	Phase 1/2	Completed	2025/2/12	B-cell CLL SLL MCL Marginal Zone Lymphoma
OTSA101	NCT04176016	Phase 1	Terminated	2024/2/2	Relapsed or Refractory Synovial Sarcoma
Foxy-5	NCT02020291	Phase 1	Completed	2016/2/2	Metastatic BC CRC PCa
Foxy-5	NCT02655952	Phase 1	Completed	2018/12/28	Metastatic BC MCRC Metastatic PCa
Foxy-5	NCT03883802	Phase 2		2022/12/9	CRC
CWP232291	NCT03055286	Phase 1/2		2021/12/13	AML
CWP232291	NCT01398462	Phase 1	Completed	2016/3/8	AML Chronic Myelomonocytic Leukemia Myelodysplastic Syndrome|Myelofibrosis
CWP232291	NCT02426723	Phase 1	Completed	2019/5/17	Multiple Myeloma
LY2090314	NCT01287520	Phase 1	Completed	2019/2/25	Advanced Cancer
LY2090314	NCT01632306	Phase 1/2	Terminated	2019/1/15	PCA
LY2090314	NCT01214603	Phase 2	Completed	2018/11/19	Leukemia
Tegavivint	NCT05797805	Phase 1/2	Recruiting	2024/8/27	Advanced HCC
Tegavivint	NCT04874480	Phase 1	Active, not recruiting	2025/1/30	Recurrent Leukemia|Refractory Leukemia
Tegavivint	NCT04851119	Phase 1/2	Recruiting	2024/10/26	Colorectal Carcinoma|Endometrial Carcinoma|Melanoma|Neuroblastoma|OC|PDAC|Recurrent Desmoid Fibromatosis/ES/HB/HCC/Malignant Solid Neoplasm/NHL/OS|Refractory Desmoid Fibromatosis/ES/HB/HCC/Malignant Solid Neoplasm/NHL/OS|Solid Pseudopapillary Neoplasm of the Pancreas|Wilms Tumor
Tegavivint	NCT04780568	Phase 1	Recruiting	2025/1/1	Metastatic NSCLC |Stage IV Lung Cancer AJCC V8|Stage IVA/B Lung Cancer AJCC V8
Tegavivint	NCT03459469	Phase 1	Completed	2023/3/1	Desmoid Tumor
PRI-724	NCT01606579	Phase 1/2	Completed	2017/8/17	AML|Chronic Myeloid Leukemia
PRI-724	NCT03620474	Phase 1/2	Completed	2024/10/10	Hepatitis C Hepatitis B Liver Cirrhoses
PRI-724	NCT01764477	Phase 1	Completed	2017/8/17	Advanced PCA Metastatic PCA Pancreatic Adenocarcinoma
PRI-724	NCT02195440	Phase 1	Completed	2022/7/7	Hepatitis C Virus-infected Cirrhosis
PRI-724	NCT01302405	Phase 1	Terminated	2017/8/17	Advanced Solid Tumors
PRI-724	NCT02828254		Completed	2022/7/7	Hepatitis C Virus-infected Cirrhosis
PRI-724	NCT02413853	Phase 2	Withdrawn	2017/4/17	Colorectal Adenocarcinoma Stage IVA CRC Stage IVB CRC
Umbralisib	NCT04163718	Phase 2	Terminated	2023/3/24	CLL
Umbralisib	NCT03364231	Phase 2	Completed	2023/6/23	Marginal Zone Lymphoma Waldenstrom Macroglobulinemia Non Follicular Indolent NHL
Umbralisib	NCT04508647	Phase 2	Completed	2024/3/21	Marginal Zone Lymphoma FL
Umbralisib	NCT03776864	Phase 2	Terminated	2023/5/3	HL
Umbralisib	NCT02742090	Phase 2	Terminated	2024/7/3	CLL
Umbralisib	NCT04149821	Phase 2	Terminated	2023/6/15	CLL SLL
Umbralisib	NCT04692155	Phase 1/2	Terminated	2023/9/28	MCL
Umbralisib	NCT03919175	Phase 2	Terminated	2024/5/8	Lymphoma FL
Umbralisib	NCT03828448	Phase 2	Terminated	2023/7/24	FL SLL
Umbralisib	NCT02793583	Phase 1/2	Terminated	2022/7/21	DLBCL FL Marginal Zone Lymphoma
Umbralisib	NCT03801525	Phase 1/2	Terminated	2024/4/19	CLL SLL
Umbralisib	NCT04783415	Phase 2	Active, not recruiting	2024/12/2	MCL
Umbralisib	NCT05152459	Phase 1/2	Withdrawn	2023/5/31	Recurrent FL Refractory FL
Umbralisib	NCT02656303	Phase 2	Terminated	2023/6/22	CLL
Umbralisib	NCT04624633	Phase 2	Active, not recruiting	2024/12/24	CLL SLL Relapsed CLL
Umbralisib	NCT02535286	Phase 1	Completed	2022/10/20	CLL Richter Syndrome
Umbralisib	NCT04635683	Phase 1	Withdrawn	2022/9/30	Recurrent B-Cell NHL Recurrent Extranodal Marginal Zone Lymphoma of Mucosa-Associated Lymphoid Tissue Recurrent FL
Umbralisib	NCT03283137	Phase 1	Active, not recruiting	2025/2/7	CLL B-cell NHL
Umbralisib	NCT03379051	Phase 1/2	Terminated	2022/8/22	CLL NHL
Umbralisib	NCT03671590	Phase 1	Terminated	2024/7/19	NHL CLL
Umbralisib	NCT04016805	Phase 2	Terminated	2023/7/24	CLL
Umbralisib	NCT02268851	Phase 1	Completed	2024/11/15	CLL/SLL MCL
Umbralisib	NCT02612311	Phase 3	Terminated	2024/12/13	CLL
Umbralisib	NCT03207256	Phase 2	Terminated	2022/7/21	CLL NHL
Vantictumab	NCT01345201	Phase 1	Completed	2020/9/9	Solid Tumors
RXC004	NCT04907851	Phase 2	Completed	2024/3/8	Advanced Solid Tumours
RXC004	NCT04907539	Phase 2	Completed	2024/5/3	CRC
Zilovertamab	NCT03833180	Phase 1	Completed	2024/1/12	CLL MCL FL
Zilovertamab	NCT04504916	Phase 2	Terminated	2024/8/15	TNBC Non-squamous NSCLC NSCLC
Zilovertamab	NCT06395103	Phase 1/2	Recruiting	2025/2/18	B-cell Acute Lymphoblastic Leukemia DLBCL Burkitt Lymphoma Neuroblastoma ES
Zilovertamab	NCT05144841	Phase 2	Active, not recruiting	2024/11/8	Relapsed or Refractory DLBCL
Zilovertamab	NCT05431179	Phase 3	Withdrawn	2023/4/21	MCL Lymphoma Lymphoproliferative Disorders Lymphatic Diseases Immunoproliferative Disorders Immune System Diseases Lymphoma, NHL
Zilovertamab	NCT05562830	Phase 1/2	Active, not recruiting	2024/11/20	Urothelial Carcinoma
Zilovertamab	NCT04501939	Phase 2	Active, not recruiting	2023/11/28	CLL
Zilovertamab	NCT05458297	Phase 2	Recruiting	2025/2/20	CLL MCL FL Richter Transformation Lymphoma
Zilovertamab	NCT05406401	Phase 2	Active, not recruiting	2024/10/15	Lymphoma, DLBCL
Zilovertamab	NCT06717347	Phase 3	Recruiting	2025/2/20	DLBCL
Zilovertamab	NCT05139017	Phase 2/3	Recruiting	2025/1/10	DLBCL

Data sources – ClinicalTrials.gov

*Abbreviations: SCC* squamous cell carcinoma,*CRC* colorectal cancer,*PCA* pancreatic cancer,*HCC* hepatocellular carcinoma,*NSCLC n*on-small cell lung cancer,*EC* esophageal cancer,*GC* gastric cancer,*GA g*astric adenocarcinoma,*OC* ovarian cancer,*BTC* biliary tract cancer,*BC* breast cancer,*TNBC* triple-negative breast cancer,*CLL* chronic lymphocytic leukemia,*SLL* small lymphocytic lymphoma,*MCL* mantle cell lymphoma,*AML* acute myeloid leukemia,*DLBCL* diffuse large B-cell lymphoma,*NHL* Non-Hodgkin lymphoma,*PDAC* Pancreatic ductal adenocarcinoma,*ES* Ewing sarcoma,*HB* hepatoblastoma,*OS* osteosarcoma,*FL* follicular lymphoma,HL Hodgkin's lymphoma,*PCa* prostate cancer,*GEC* gastroesophageal cancer,*mCRC* metastatic colorectal cancer

### Preclinical studies and clinical trials of WNT pathway inhibitors

Numerous preclinical studies have demonstrated that targeting components of the WNT signaling pathway can effectively inhibit the activation of the WNT signaling pathway in cancer. Furthermore, a large number of related clinical trials have reported promising anticancer activity. Below, we describe WNT signaling inhibitors according to the components targeting the signaling pathway.

#### PORCN inhibitors

WNT palmitoylation is crucial for the activation of its signaling pathway. As an O-acyltransferase localized in the endoplasmic reticulum, PORCN catalyzes the binding of palmitoleic acid to the Ser residue of WNT proteins, thereby mediating the palmitoylation modification of the ligand. Therefore, inhibiting PORCN can prevent the secretion of WNT proteins into the extracellular matrix, thereby reducing the excessive accumulation of β-catenin in the cytoplasm to promote the expression of cancer-related genes. Currently, various PORCN inhibitors have been in preclinical studies and clinical trials. For instance, in OC, upregulation of the WNT/β-catenin pathway is associated with tumor proliferation and chemotherapy resistance. WNT974 (LGK974) blocks the WNT pathway activity by inhibiting PORCN, leading to cell cycle arrest in ascites cells rather than direct cytotoxicity [216]. Additionally, WNT974 has been validated to be effective in head and neck squamous cell carcinoma with aberrant WNT signaling [217]. Several clinical trials have investigated its potential. In a Phase Ib dose-escalation study (NCT02228120), researchers investigated the combination of WNT974 with encorafenib and cetuximab in patients with The B-Raf proto-oncogene (BRAF) V600E-mutant metastatic CRC (mCRC) harboring RNF43 mutations or R-spondin (RSPO) fusions. As a secreted glycoprotein, RSPO binds to LGR4/5/6 receptors and significantly potentiates WNT/β-catenin signaling pathway. RSPO fusions have been demonstrated to drive hyperactivation of WNT/β-catenin signaling in multiple cancer types, serving as an oncogenic driver. In the 10 mg WNT974 cohort, bone toxicity occurred in 45% of patients. Although the disease control rate reached 85%, the overall response rate was only 10%, with most patients achieving stable disease. Compared to the historical encorafenib and cetuximab dual regimen, the triple-combination therapy did not demonstrate significantly enhanced antitumor activity. Due to the high incidence of bone toxicity and lack of superior efficacy over the dual regimen, the study was terminated after Phase Ib, and no Phase II trial was initiated. Another Phase I study (NCT01351103) demonstrated that WNT974 monotherapy was well-tolerated in patients with advanced solid tumors. The most common adverse event was dysgeusia (50% of patients), mostly Grade 1–2. Notably, AXIN2 mRNA expression was significantly reduced in 94% of skin biopsies and 74% of tumor biopsies, confirming effective WNT pathway suppression. Despite these findings, WNT974 monotherapy showed limited antitumor efficacy. ETC-159 is an oral PORCN inhibitor that blocks the palmitoylation of WNT proteins to inhibit the secretion and activity of WNTs. ETC-159 can effectively suppress aberrant WNT signaling in CRC and OS [218, 219]. Furthermore, in the treatment of RNF43-mutant PCA, the combination of ETC-159 and the pan-PI3K inhibitor GDC-0941 effectively inhibits the growth of xenografts in vivo [220]. In 2016, ETC-159 was reported in a Phase 1, first-in-human dose-escalation study (NCT02521844) for solid tumors, evaluating its safety, tolerability, maximum tolerated dose (MTD), pharmacokinetics (PK), and pharmacodynamics. The Phase 1 study demonstrated that ETC-159 had a manageable safety profile and preliminarily validated its inhibitory effect on the WNT pathway, though its single-agent antitumor activity was limited. The study also assessed whether ETC-159, at a dose of 24 mg every other day combined with prophylactic denosumab treatment, resulted in toxicities such as compression fractures, hyperbilirubinemia, and elevated serum b-CTX, which were considered dose-limiting toxicities (DLT) of ETC-159. Subsequently, the results of a Phase Ib dose-escalation study evaluating ETC-159 in combination with pembrolizumab, a programmed death (PD)−1 inhibitor, for the treatment of advanced or metastatic solid tumors were reported. CGX1321 is an orally bioavailable small molecule PORCN inhibitor. In OC, CGX1321 significantly reduces tumor burden, improves survival rates, and the functions of T cells, macrophages, and dendritic cells by inhibiting WNT secretion, transforming the tumor microenvironment into an "immune-hot" state [221]. To enhance the efficacy of CGX1321, it has been encapsulated in liposomes, successfully achieving efficient targeted delivery to tumor tissues while significantly reducing toxicity to normal tissues [222]. Currently, there are two clinical studies underway: a Phase I dose-escalation and dose-expansion study of CGX1321 monotherapy, and a study of CGX1321 in combination with pembrolizumab. The primary objectives are to evaluate the safety, tolerability, and recommended dose of CGX1321, as well as to characterize its antitumor activity, PK profile, and PD responses. In terms of safety, CGX1321—both as monotherapy and in combination—demonstrated a manageable side effect profile, with most adverse events being Grade 1–2 (e.g., mild dysgeusia). Bone resorption could be mitigated with prophylactic denosumab or zoledronic acid. As a monotherapy, CGX1321 achieved disease stabilization in 71% of colorectal or small intestinal cancer patients harboring RSPO fusions or RNF43mutations, with a median progression-free survival of 112 days. In the combination therapy arm, three patients with RSPO3-fusion tumors achieved partial responses after treatment with CGX1321 plus pembrolizumab. In addition, a small molecule compound 1 has been reported. It is capable of simultaneously inhibiting the WNT and Hedgehog signaling pathways. Mechanistic studies indicate that compound 1 achieves dual inhibition by targeting PORCN and SMO which belongs to the FZD family of seven-transmembrane proteins with a typical G protein-coupled receptor structure and can mediate the activation of the Hedgehog pathway through phosphorylation of its intracellular C-terminal domain. The amino group in the right tricyclic structure of compound 1 is crucial for Hedgehog inhibition, while the fluorine atom on the left aromatic ring significantly impacts WNT inhibition [223]. Moreover, a novel PORCN inhibitor, WHN-88, can effectively inhibit the palmitoylation of WNT ligands through its unique diiodopyridone structure [224]. Other PORCN inhibitors, including Wnt-c59, pyridylacetamide derivative GNF-6231, and RXC004, have demonstrated therapeutic anti-tumor activity in various cancers [225–227]. RXC004 primarily inhibits PORCN O-acyltransferase and demonstrated favorable safety and tolerability in a Phase I study (NCT03447470) involving patients with advanced solid tumors. Subsequently, between March 24, 2021, and June 30, 2022, 14 unselected patients with advanced solid tumors received RXC004 at doses of 1 mg and 1.5 mg once daily (QD) in combination with nivolumab. The adverse event profile of the combination therapy was generally consistent with that of RXC004 monotherapy, and disease control was observed in some patients in the 1.5 mg QD combination cohort. Following the completion of the Phase I study (NCT03447470), further evaluation of RXC004 was conducted in patients with metastatic microsatellite-stable CRC harboring RNF43mutations or RSPO fusions (NCT04907539). The monotherapy arm primarily assessed the 16-week disease control rate, while the combination arm with nivolumab focused on objective response rate. Another Phase II study (NCT04907851) is evaluating the efficacy of RXC004 in BTC and RNF43-mutant PCA.

#### FZD Inhibitors

FZD has emerged as pivotal molecules in cancer research, exerting pleiotropic effects on cancer progression, including modulation of tumor growth, metastasis, drug resistance, and CSC properties. Accumulating studies suggest that overexpressed FZD exerts extensive oncogenic effects through the WNT signaling pathway. Given the crucial regulatory role of FZDs in tumors and their favorable localization on the cell membrane, they have been identified as promising therapeutic targets for cancer treatment. OMP-54F28 (Ipafricept) is a fusion protein consisting of the extracellular domain of FZD8 and IgG1 Fc, which blocks WNT signaling by competitively binding to WNT ligands. Preclinical studies have shown that OMP-54F28 monotherapy significantly inhibits tumor growth, reduces the frequency of CSCs and diminishes metastasis [228]. To date, three Phase 1b studies have been conducted, including evaluating OMP-54F28 in combination with nab-paclitaxel and gemcitabine for PCA (NCT02050178), carboplatin and paclitaxel for OC (NCT02092363), and sorafenib for HCC (NCT02069145). Despite promising therapeutic efficacy, bone toxicity has halted the further development [80]. OMP-18R5 (Vantictumab) is initially a monoclonal antibody targeting FZD7. Subsequently, OMP-18R5 was found to bind to FZD1/2/5/7/8 and to block the WNT3A signaling pathway [229]. In the first-in-human Phase I trial (NCT01345201), OMP-18R5 demonstrated manageable safety, clear WNT pathway inhibition, and preliminary clinical activity. However, one patient experienced a Grade 2 compression fracture. Preclinical studies suggested enhanced antitumor effects when OMP-18R5 was combined with paclitaxel. In a Phase Ib clinical trial (NCT01973309), 48 patients with locally advanced or metastatic human epidermal growth factor receptor 2 (HER2)-negative BC (45.8% hormone receptor (HR)-positive, 54.2% triple-negative), with a median age of 54 years (66.6% previously treated with metastatic chemotherapy), were enrolled. Multiple cohorts received OMP-18R5 at escalating doses from 3.5 mg/kg (every 2 weeks) up to 8 mg/kg (every 4 weeks) in combination with weekly paclitaxel (90 mg/m^2^). Despite promising efficacy, serious adverse events—particularly fractures—limited further development of OMP-18R5 in metastatic BC. Another study (NCT02005315) showed that OMP-18R5 was well-tolerated in the Phase Ia trial, though bone-related toxicity was observed. In the Ib dose-escalation phase, OMP-18R5 was administered intravenously every two weeks alongside nab-paclitaxel (125 mg/m^2^) and gemcitabine (1000 mg/m^2^). The regimen was later adjusted to a four-week schedule with the addition of zoledronic acid as a preventive measure. This modified dosing strategy effectively reduced fracture risk. Based on structural similarities with the SMO receptor, researchers constructed a homology model of FZD7 transmembrane domain (TMD), revealing two conserved binding pockets composed of hydrophobic residues in the transmembrane region. These binding sites have been targeted for drug design. Molecular docking studies indicate that SRI37892 occupies the hydrophobic core at the bottom of the TMD through a benzimidazole fragment, forming π-π interactions, while its dihydroquinolinone moiety binds to the top hydrophobic region and interacts with Glu492 via hydrogen bonds, enhancing binding stability. SRI37892 exhibits potent efficacy in TNBC cells [230]. In addition to targeting the transmembrane domain of FZD, inhibitors specifically binding to the extracellular cysteine-rich domain (CRD) have been reported. Fz7-21, as the first small molecule ligand targeting the FZD subclass, specifically binds to the CRD and locks the FZD7 in an inactive state by inducing CRD dimerization and altering the conformation of its lipid-binding groove [231]. On this basis, researchers successfully optimize the peptide Fz7-21 by combining structure-based rational design and phage display technology, significantly enhancing its binding affinity and selectivity. Key optimization strategies include the deletion of two N-terminal residues (Δ2), critical amino acid substitutions (e.g., S3T, L6Tba, F8F(3-Cl), V12I), and mutations obtained from phage screening (e.g., E7K, F8L). The final peptides (e.g., peptides 32 and 34) effectively block the binding of Clostridium difficile toxin B (TcdB) to FZD7 and inhibit the pathogenicity of TcdB by inducing receptor dimerization to create steric hindrance [232]. Additionally, the ligand zinc05972969 can also target the CRD of FZD7, providing a new direction for the treatment of TNBC [233]. Employing a structure-based drug design approach, researchers conducted virtual screening of 10,000 plant-derived compounds and ultimately identified that Cycloatrobiloxanthone (CAX) exhibits high binding affinity to the FZD7-CRD domain. CAX not only demonstrates promising anticancer activity in the treatment of GBM but also possesses favorable oral bioavailability, low toxicity, high gastrointestinal absorption capacity, and the potential to cross the blood–brain barrier [234]. Other inhibitors targeting FZD7 include anti-FZD7 single-chain antibodies (scFv-I and scFv-II), which bind to key regions of FZD7 involved in WNT ligand binding through epitope design [235]. Researchers also conjugate FZD7 antibodies to gold shell nanoparticles (FZD7-NS), significantly enhancing the binding affinity of the antibodies to FZD7 on the surface of TNBC cells by leveraging the multivalent binding effect of nanoparticles [236]. Currently, targeting FZD7 represents a promising therapeutic strategy characterized by low drug toxicity and high anticancer activity. Additionally, compounds such as NSC36784 and NSC654259 effectively inhibit the WNT3A-induced β-catenin signaling pathway [237]. For FZD10, OTSA101 is a humanized monoclonal antibody targeting FZD10. OTSA101-DTPA-90Y is labeled with a β-radiation-emitting yttrium Y90 for OTSA101. OTSA101-DTPA-90Y selectively kills cancer cells overexpressing FZD10 [238]. In addition, some inhibitors exhibit pan-specificity, targeting multiple FZDs. F2.A is a newly developed antibody targeting six out of the ten human FZDs (FZD1/2/4/5/7/8). Researchers first identified a specific anti-FZD antibody (F2) that matches OMP-18R5, and then developed a variant F2.A with specificity for FZD4 using combinatorial antibody engineering. According to their research, F2.A can selectively bind to FZD4 without competing with FZD4-specific ligand Norrin [239]. Additionally, there is an F2 scFv corresponding to the FZD1/2/7 CRD [240].

#### LRP5/6 inhibitors

LRP5/6 forms a trimer with FZD and WNT, thereby initiating the WNT signaling pathway. Its phosphorylation plays a crucial role in the activation of the pathway. For example, salinomycin inhibits the WNT signaling pathway by interfering with LRP6 phosphorylation [241]. The proton pump inhibitor pantoprazole can also inhibit the proliferation of GC cells and induce apoptosis by reducing the levels of phosphorylated LRP6 [242]. In addition, an anthelmintic drug niclosamide has been shown to effectively inhibit the expression of LRP6, leading to a reduction in the nuclear accumulation of β-catenin. Currently, niclosamide has demonstrated significant anti-tumor effects in PCa, BC, and CRC [243]. In PCa, MESD is a specific chaperone protein responsible for promoting the folding of the LRP family. MESD inhibits the phosphorylation of LRP5/6 to reduce β-catenin accumulation by binding with high affinity to mature LRP5/6 on the cell surface, thereby inhibiting the proliferation of PCa cells [244]. In addition, through pharmacophore model-guided virtual screening, molecular docking simulations and in vitro experiments, Choi, J et al. first discovered that the quinoxaline derivative BMD4503-2 can serve as a novel inhibitor of the LRP5/6-sclerostin interaction, directly targeting the key node of the WNT/β-catenin signaling pathway that regulates bone homeostasis. BMD4503-2 competitively binds to the E1 domain of LRP5/6, disrupting the interaction between sclerostin and LRP5/6 [245]. In addition, DKN-01, the cholesterol-depleting agent MβCD, and the vitamin D receptor agonist calcitriol can also inhibit tumors by reducing LRP6 protein levels [246, 247].

#### DVL inhibitors

DVL is a crucial component of the WNT signaling pathway. DVL recruits the destruction complex to the cell membrane and binds to the cytoplasmic carboxyl terminus of FZD proteins via its PDZ domain. FJ9 is a novel small molecule inhibitor that suppresses the canonical WNT signaling pathway by specifically blocking the interaction between the FZD7 and the PDZ domain of DVL [248]. Additionally, NSC668036 and 3289–8625 can also inhibit WNT signaling by binding to the PDZ domain of DVL [249]. DVL2 is significantly overexpressed in 90% of tumor tissues from hepatoblastoma (HB) patients. Knockdown of DVL2 via shRNA significantly inhibits the proliferation and invasive capabilities of HB cell lines, indicating that DVL2 plays a pro-oncogenic role in HB. Niclosamide can downregulate the expression of DVL2 and β-catenin in a dose-dependent manner, thereby inhibiting the proliferation of HB cells [250].

#### TNKS inhibitors

TNKS belong to the Poly (ADP-ribose) polymerase (PARPs) family, with two subtypes: TNKS1 (PARP5a) and TNKS2 (PARP5b). TNKS-mediated PARsylation of AXIN induces its degradation via the ubiquitin–proteasome pathway, and subsequent AXIN degradation triggers the release of β-catenin from the destruction complex into the nucleus. Therefore, TNKS inhibitors typically stabilize AXIN, thereby exerting anti-tumor effects. The TNKS inhibitor IWR-1 inhibits the WNT/β-catenin pathway by stabilizing AXIN protein, significantly reducing the viability of OS CSCs, inducing cell cycle arrest and triggering mitochondrial-dependent apoptosis. The combination of IWR-1 with doxorubicin exhibits synergistic cytotoxicity, significantly enhancing the sensitivity of CSCs to doxorubicin [251]. In addition, a small molecule compound XAV939 significantly increases AXIN protein levels by inhibiting the activity of TNKS 1 and TNKS 2, thereby enhancing the phosphorylation and degradation of β-catenin [252]. XAV939 and IWR-1 also can inhibit the proliferation of LCA cells in a dose-dependent manner [253]. Additionally, TNKS inhibitors such as G007-LK, G244-LM, MSC2504877, JW74, RK-287107, LZZ-02, JW55, and AZ1366 can significantly reduce nuclear β-catenin levels by stabilizing AXIN protein [254–260]. Researchers also discover that USP7 directly interacts with AXIN through its N-terminal TRAF domain and relies on its deubiquitinating enzyme activity to remove polyubiquitin modifications from AXIN. This process prevents AXIN from being degraded by the proteasome, thereby stabilizing the AXIN protein and negatively regulating the WNT/β-catenin signaling pathway [261]. STP1002 is a novel oral selective inhibitor of TNKS. It demonstrates good efficacy in APC-mutated CRC [262].

#### CK1 Agonists

CK1 is a crucial component of the destruction complex and plays a pivotal role in preventing the nuclear localization of β-catenin, indicating that activating CK1 is an attractive therapeutic target. For instance, Pyrvinium, an FDA-approved drug, can directly bind to CK1α, enhancing its kinase activity through allosteric effects to promote the phosphorylation and degradation of β-catenin [263]. Additionally, Pyrvinium stabilizes CK1α protein levels by inhibiting the E3 Ubiquitin Ligase Cereblon (CRBN) -mediated degradation which plays a role in substrate recognition within the CRL4^CRBN^ E3 ubiquitin ligase complex, further suppressing WNT signaling [264]. Moreover, SSTC3, a small molecule CK1α activator, inhibits the WNT signaling pathway through CK1α activation. Its pharmacokinetic properties are superior to earlier drugs (e.g., Pyrvinium), maintaining effective concentrations in vivo and significantly inhibiting the proliferation of CRC cells and tumor growth [265].

#### β-catenin/TCF transcriptional complex inhibitors

Currently, several compounds have been reported to block the β-catenin/TCF complex. For example, the researchers have successfully developed a cell-penetrating stabilized peptide inhibitor named NLS-StAx-h. By integrating the design strategy of cell-penetrating peptides with peptide stabilization technology, they significantly enhance the cellular uptake efficiency and targeting capability of the peptide. This inhibitor effectively suppresses the WNT signaling pathway by specifically blocking the interaction between β-catenin and TCF/LEF transcription factors. Moreover, the intracellular distribution and stability of the peptide were significantly improved through the optimization of the nuclear localization sequence and arginine derivatives [266]. In addition, Tumor necrosis factor receptor-associated factor 2 (TRAF2), a member of the TRAF superfamily of proteins, regulates NF-κB and MAPK signaling pathways through its E3 ubiquitin ligase activity. TRAF2 positively regulates the WNT/β-catenin signaling pathway by directly interacting with the N-terminus of β-catenin through its TRAF-C domain, promoting CRC cell proliferation and tumor growth. Liquidambaric acid (LDA) directly targets the TRAF-C domain of TRAF2, disrupting the binding between TRAF2 and β-catenin, thereby inhibiting WNT signaling activity. LDA also blocks the formation of the TRAF2/β-catenin/TCF4/TNIK complex, which maintains the transcriptional activity of WNT signaling in CRC cells [267]. Demethylsteroid A3 (Sdy-1) is a sterol isolated from the endophytic fungus HQD-6 of the Rhizophora genus. Sdy-1 can significantly inhibit the proliferation, migration, and invasion of liver and CC cells, induce apoptosis, and suppress tumor growth by arresting the cell cycle at the G1 phase. Molecular docking experiments reveal that Sdy-1 binds to key sites such as THR-433 of the β-catenin protein. Sdy-1 can directly reduce the mRNA level of β-catenin and block the binding of nuclear β-catenin to TCF/LEF transcription factors, thereby inhibiting the expression of downstream target genes [268]. Panobinostat (LBH589), as a pan-histone deacetylase inhibitor, exerts significant anti-tumor effects in BC treatment by upregulating the expression of the tumor suppressor gene APCL to inhibit the WNT/β-catenin signaling pathway. Panobinostat activates the transcription of APCL by increasing histone acetylation levels and the upregulation of APCL promotes the ubiquitination and degradation of β-catenin [269]. Luteolin, a flavonoid monomer isolated from Paulownia flowers, binds to β-catenin through hydrogen bonds, causing conformational changes (reduced α-helix, increased β-sheet) to inhibit its activity [269]. 4β-hydroxywithanolide E (4βHWE) is a natural glycolic acid with a 17α-oriented side chain. 4βHWE promotes the phosphorylation of β-catenin at Ser33/Ser37/Thr41 sites to enhance its ubiquitination and degradation [270]. In addition, researchers identified a small molecule inhibitor C2, which directly targets the allosteric site of β-catenin, alters its conformation, and exposes phosphorylation sites, triggering the degradation of β-catenin via the ubiquitin–proteasome pathway [271]. B-cell lymphoma 9 (BCL9) is a β-transcriptional co-activator. The C-1 inhibits the β-catenin/BCL9 complex, thereby blocking the WNT signaling pathway [272]. Moreover, researchers isolated a novel compound, Linderapyrone, from the stems and branches of Lindera umbellata. Experiments show that Linderapyrone, with its pyrone and monoterpene moieties, can effectively block the WNT signaling pathway by inhibiting β-catenin/TCF transcriptional activity [273]. Additionally, small molecule inhibitors of the β-catenin/TCF transcriptional complex, PKF115-584, CGP049090, and PKF118-310, have been shown to significantly inhibit the progression of HCC [274, 275]. In another study, it was found that CGP049090 and PKF115-584 effectively kill CLL cells but exhibit lower toxicity to normal B cells, demonstrating selective anti-cancer activity [276]. Furthermore, researchers identified the compound ZINC02092166 through high-throughput screening, which effectively inhibits β-catenin/TCF. To address the potential reactivity of the acyl hydrazone group that may cause off-target effects, derivatives 26, 33, and 37 were designed by replacing the acyl hydrazone group with chemically more stable structures such as amides or tetrazole rings. These compounds maintain inhibitory activity while significantly reducing off-target effects and enhancing selectivity for β-catenin/TCF [277].

CREB-binding protein (CBP) is a crucial transcriptional coactivator, essential for activating cancer-related WNT genes. ICG-001, a selective CBP/β-catenin antagonist screened from 5000 secondary structure mimics, specifically binds to CBP, thereby blocking the interaction between CBP and β-catenin. This drug forces CSCs to switch from self-renewal to symmetric differentiation, thereby eliminating CSCs, while normal somatic stem cells, which rely on asymmetric division to maintain homeostasis, are not significantly affected [278]. Moreover, the combination of ICG-001 and ZSTK-474 (a PI3K inhibitor) significantly enhances the cytotoxicity of ICG-001, inducing apoptosis in T-ALL [279]. ICG-001 also exerts anticancer effects in pancreatic ductal adenocarcinoma (PDAC) and tamoxifen-resistant BC cells [280, 281]. PRI-724 is a second-generation small molecule antagonist of CBP/β-catenin interaction, which inhibits the activity of the WNT/β-catenin/CBP signaling pathway by blocking the binding of β-catenin to CBP, thereby reversing chemotherapy resistance caused by RBMS3 deficiency. In RBMS3-deficient EOC cells, the combination of PRI-724 and cisplatin significantly enhances chemotherapy sensitivity and apoptosis rates [282]. In a Phase I/IIa study (NCT03620474), PRI-724 administered at a dose of 280 mg/m^2^ over 4 h (twice weekly via intravenous infusion for 12 weeks) was preliminarily assessed as well-tolerated in patients with liver cirrhosis. Based on liver biopsy evaluations (collagen proportionate area or fibrosis staging), PRI-724 did not significantly reduce hepatic fibrosis area. However, significant improvements were observed in liver stiffness measurement and serum albumin levels after treatment. Through fragment-based virtual screening and structure-guided medicinal chemistry optimization, highly effective and selective compounds (1-(1H-indol-1-yl) ethanone derivatives) are identified. Among them, derivative 32 h exhibits twice the inhibitory activity against the CBP bromodomain compared to the known inhibitor SGC-CBP30 and shows significant selectivity for CBP/EP300 [283]. Other inhibitors include GNE-781, JW67, JW74, and E7386 [284–287].

### Advances in WNT-targeted therapies

New therapeutic approaches targeting the WNT signaling pathway are being explored, which may offer new possibilities for enhancing anticancer activity and reducing adverse reactions in future clinical trials, including the combination of WNT signaling pathway targeting with immunotherapy, Antibody–Drug Conjugation, non-coding RNA therapeutics, Antisense Oligonucleotides, Proteolysis Targeting Chimeras and Molecular glues.

#### Immunotherapy

Deactivation of the immune system constitutes a critical mechanism underlying cancer resistance to immune checkpoint inhibitors. Reactivating the immune system has emerged as a promising antitumor strategy. Currently, multiple studies have shown that the WNT signaling pathway is involved in mediating the regulation of the immune system in cancer. Consequently, combining WNT/β-catenin pathway inhibitors with immunotherapy may overcome therapeutic resistance and potentiate the antitumor capacity of immune cells. Ganesh, S et al. developed DCR-BCAT, a therapeutic agent employing RNA interference to specifically suppress β-catenin expression. DCR-BCAT remarkably enhances T cell and dendritic cell infiltration in tumor microenvironment, reverses immune "cold" phenotype, and synergizes with anti-PD-1/CTLA-4 checkpoint inhibitors [288]. Another study demonstrates that in patients resistant to PD-1/PD-L1 antibodies, DKN-01 exerts anti-tumor effects primarily through immune activation in combination with pembrolizumab and nivolumab. The tumor suppression effect of DKN-01 combined with anti-PD-1 antibodies is significantly superior to that of monotherapy [289, 290]. Currently, clinical studies on the combination of WNT signaling pathway inhibitors with immune checkpoint inhibitors are increasing, which may provide new insights for enhancing the efficacy of immunotherapy and overcoming immune resistance in cancer treatment in the future.

#### Antibody-Drug Conjugation

Antibody–Drug Conjugation (ADC) utilizes antibodies to specifically recognize tumor surface antigens, enabling targeted delivery of cytotoxic agents to cancer cells. Through endocytosis and lysosomal release mechanisms, these conjugates achieve precise drug activation, significantly reducing the off-target toxicity of traditional chemotherapy [291]. Septuximab vedotin (F7-ADC) employs its antibody component to bind the extracellular domain of FZD7, facilitating drug internalization into lysosomes and subsequent release of the cytotoxic payload monomethyl auristatin E (MMAE), thereby selectively eliminating OC cells with high FZD7 expression. Notably, F7-ADC does not interfere with the WNT signaling pathway itself, circumventing the bone-related adverse effects commonly observed with conventional WNT inhibitors [292]. Other ADCs include PF-06647020, LGR5-mc-vc-PAB-MMAE, and LRG5-NMs818 [293, 294].

#### Non-coding RNA therapeutics

MiRNAs are a type of non-coding RNA that primarily function by interacting with mRNAs. Substantial evidence demonstrates that miRNAs exhibit tumor-suppressive functions in cancers characterized by aberrant activation of the WNT signaling pathway. These regulatory RNAs effectively modulate protein levels in the WNT/β-catenin pathway, thereby suppressing the expression of molecules associated with tumorigenesis and progression [295]. For instance, miR-384-5p exerts anti-tumor effects by downregulating the expression of FZD1, FZD2, and LRP6 [296]. In addition, another type of non-coding RNA, siRNA, also exhibits anti-tumor potential. Researchers have engineered a novel bioengineered BERA-WNT5A siRNA that inhibits the non-canonical WNT pathway through WNT5A/FZD2 axis suppression, effectively curbing the growth of therapy-resistant PCa [297].

#### Antisense Oligonucleotides

Antisense Oligonucleotides (ASOs) are synthetic short-chain nucleic acid molecules that selectively target specific RNA or DNA sequences through Watson–Crick base pairing principles. Their primary mechanism involves binding to target mRNA via complementary base pairing, forming duplex structures that either trigger mRNA degradation or block its translation into proteins. ASOs can specifically target oncogenes or modulate aberrant splicing sites, thereby inhibiting tumor growth, angiogenesis, or metastasis [298]. In one study, lncRNA AC104041.1 is significantly overexpressed in head and neck squamous cell carcinoma. LncRNA AC104041.1 sponges miR-6817-3p, thereby relieving the inhibitory effect of miR-6817-3p on WNT2B, driving tumor growth and metastasis. ASO targeting AC104041.1 effectively inhibits tumor growth and metastasis, while the antibiotic salinomycin further enhances the anti-tumor effect of the ASO by blocking the WNT/β-catenin signaling pathway [299]. Researchers also used second-generation ASOs to target β-catenin, reducing β-catenin mRNA levels by 70–80% and cytoplasmic/nuclear protein levels in the liver and white adipose tissue, thereby regulating lipid metabolism [300].

#### Proteolysis Targeting Chimeras

Proteolysis Targeting Chimeras (PROTACs), as an emerging drug development technology, catalyze the degradation of target proteins through the ubiquitin–proteasome system, providing new insights for the treatment of cancer and other diseases. PROTACs are bifunctional small-molecule drugs composed of three parts: (1) a target protein ligand that specifically binds to the target protein; (2) an E3 ubiquitin ligase; and (3) a linker that optimizes the spatial conformation between the two, inducing the proximity of the target protein and the E3 ligase [301]. Currently, researchers have applied PROTACs to target the WNT signaling pathway for cancer treatment. By conjugating the β-catenin-binding peptide xStAx with a VHL E3 ligase ligand, the designed PROTAC peptide xStAx-VHLL achieves sustained degradation of β-catenin through the ubiquitin–proteasome system, with effects significantly superior to those of the xStAx peptide alone or existing small-molecule inhibitors of the WNT signaling pathway (e.g., PRI-724, PNU-74654). However, PROTACs face limitations in binding capacity, specificity, and availability [302]. Therefore, based on the co-crystal structure of the BCL9 protein and β-catenin, researchers design a peptide-based degrader called PepTAC (βCatPepTAC). Utilizing the efficient cellular uptake, stability, and targeting capabilities of lipid nanoparticle carriers, NP-delivered βCatPepTAC significantly degrades β-catenin and inhibits WNT-driven transcriptional activity [303].

#### Molecular glues

Molecular glues are a class of single-molecule small compounds that reshape the interface of E3 ubiquitin ligases to induce the formation of stable ternary complexes with disease-causing proteins.This process triggers the degradation of the target proteins via the ubiquitin–proteasome pathway. Unlike bifunctional PROTACs, molecular glues do not require linking two ligands. Instead, they directly enhance the interaction between the E3 ligase and the target protein [304]. Based on the 6-trifluoromethylpyridone and biarylamide scaffolds, compounds such as NRX-252114 and NRX-252262 have been designed as molecular glues. These compounds strengthen the interaction between β-catenin and the SCFβ-TrCP E3 ligase, forming a stable ternary complex. The molecular glues promote the ubiquitination of β-catenin, leading to its degradation through the proteasome pathway, thereby exerting anti-cancer effects in CRC [305, 306].

## Challenges and future directions

The development of WNT-targeted therapies confronts a tripartite challenge spanning biological complexity, clinical stratification, and therapeutic integration. First, the dual role of WNT signaling in tissue homeostasis and tumorigenesis necessitates precision targeting to avoid catastrophic on-target toxicity, compounded by pathway redundancy and spatial heterogeneity within tumor niches. Second, biomarker innovation must transcend static genomic markers toward dynamic readouts: single-cell spatial transcriptomics could map WNT activity gradients, while liquid biopsy-based DKK1/LGR5 tracking may predict adaptive resistance. Third, rational combination strategies require systems-level optimization—WNT inhibitors with ICBs could reverse T-cell exhaustion via β-catenin/PD-L1 crosstalk blockade, while radiotherapy synergy might exploit WNT-mediated DNA repair vulnerability. Future success hinges on organ-specific pathway mapping and microenvironment-adapted delivery systems.

### Challenges of targeting the WNT signaling pathway

The WNT signaling pathway plays a critical role in maintaining normal cellular physiological functions. Aberrant activation or suppression of this pathway is closely associated with cancer initiation and progression. Preclinical studies have demonstrated that targeting the WNT pathway exhibits promising antitumor activity, indicating its key regulatory components as attractive therapeutic targets in malignancies. Therefore, researchers have devoted considerable efforts to developing drugs targeting the WNT signaling pathway [307]. Although several WNT signaling inhibitors have shown favorable outcomes in ongoing clinical trials, no WNT-specific therapeutic agent has received clinical approval to date. A major concern is that targeting the WNT signaling pathway in cancer treatment may induce adverse events. Because the pleiotropic functions of WNT signaling in normal cellular processes are indispensable for tissue homeostasis and repair, pathway inhibition may lead to unintended consequences on normal tissue functions [307]. For example, the first-generation WNT pathway inhibitors, PORCN inhibitors, such as LGK974 and ETC-159, are pan-WNT pathway inhibitors. When these two PORCN inhibitors are administered to mice, the treatment induces dose-dependent adverse effects, such as reductions in bone volume and density [308]. Another example of WNT pathway inhibitor toxicity is TNKS inhibitors. Despite exhibiting excellent antitumor efficacy in preclinical studies, current TNKS inhibitors manifest multiorgan toxicity, particularly gastrointestinal toxicity, during clinical trials [309]. In addition, studies have shown that inhibiting the WNT pathway may adversely affect normal WNT-dependent stem cell populations. Therefore, next-generation WNT inhibitors must achieve potent antitumor efficacy while preserving physiologically essential WNT signaling. Some researchers believe that the WNT signaling pathway receptor FZD is promising therapeutic targets. On one hand, the FZDs family consists of 10 members, which can be further divided into four subfamilies based on their sequences: FZD1/2/7, FZD4/9/10, FZD5/8, and FZD3/6. FZD exhibits distinct tissue-specific expression patterns and plays a crucial role in initiating multiple WNT signaling branches. On the other hand, in mouse models, FZD7 has been shown to be essential for maintaining intestinal and atrial gastric epithelial cells. FZD7 knockout mice are fertile under basic, non-challenging conditions, indicating that the loss of FZD7 in a developing organism can be compensated for by other mechanisms. Furthermore, in adults, the downregulation of FZD7 does not prevent but rather delays intestinal regeneration [310]. Although the broad-spectrum FZD inhibitor Vantictumab was discontinued in Phase I clinical trials due to an increased frequency of fractures, the lack of FZD specificity—particularly targeting FZD1, FZD4, and FZD8, which are important for bone homeostasis—is a critical factor. Therefore, the development of highly selective inhibitors targeting specific FZD subtypes is a future direction [311]. Zhang, M. et al. have proposed how the widespread presence of drug-targetable allosteric sites in FZD could guide structure- or mechanism-based drug design, and have outlined the therapeutic potential of developing bispecific ligands targeting this receptor family in the future [312]. In addition, mapping the organ-specific pathway is another noteworthy approach. A recent study utilizes machine learning models to quantify T1 relaxation times (a biomarker of interstitial fibrosis) in the liver, pancreas, heart, and kidneys among 43,881 UK Biobank participants. The research revealed both organ-specific and shared cross-organ fibrosis pathways. Through genome-wide association studies, the investigation identified genetic loci associated with fibrosis across these organs, including SLC39A8, HFE, and ABO [313].

### Development of WNT pathway biomarkers for patient stratification

The use of biomarkers for the precise stratification of cancer patients has been demonstrated to effectively reduce cancer-related mortality. These biomarkers assist clinicians in selecting appropriate treatment strategies and identifying patients at high or low risk of tumor progression, which allows for the refinement of monitoring and the allocation of early therapeutic interventions. Given that the expression of various components of the WNT signaling pathway is deregulated in many tumor tissues and contributes to tumor proliferation, invasion, and metastasis, the WNT signaling pathway may serve as a potential biomarker [311] (Table 2).

**Table 2:** The Summary of WNT Signaling Pathway Components as Cancer Biomarkers

Biomarker Type	WNT Signaling Pathway Component	Cancer Type	Role	References
Prognostic Biomarker	WNT5A	HCC	Tumor-suppressive effects	[314]
	β-catenin	PDAC	Promotes metastasis and invasion	[315]
	β-catenin	EC	Inhibits invasion and metastasis	[316]
	WNT8B	HCC	Promotes cancer cell proliferation	[317]
	β-catenin	Endometrioid Endometrial Cancer	Promotes cancer invasion	[318]
	TCF7	GC	Promotes metastasis and invasion	[319]
	WNT5A-S	CRC	Inhibits CRC cell proliferation	[320]
	WNT5A-L	CRC	Promotes CRC cell proliferation	[320]
	WNT5A	OC	Promotes migration and invasion	[321]
	WNT6	CRC, GC, BC, GBM	Promotes metastasis and invasion	[322]
	β-catenin	CC	Promotes infiltration	[323]
	WNT11	CC	Promotes metastasis	[323]
Diagnostic Biomarker	WNT11	SCLC	Promotes proliferation and EMT	[324]
	WNT3A	HCC	Promotes proliferation and differentiation	[325]
	β-catenin	Non-Seminomatous Germ Cell Tumor	Suppresses tumor immunity	[326]
	β-catenin	Desmoid-Type Fibromatosis		[327]
	LEF1	BA, Pancreatic Solid Pseudopapillary Neoplasm		[327]
	LEF1	Sinonasal Hemangiopericytoma		[328]
	β-catenin	OSCC	Promotes EMT	[329]
	APCL	OSCC	Promotes EMT	[329]
	WNT6	OS		[330]
Therapeutic Biomarker	PORCN	OC	High PORCN expression correlates with sensitivity to WNT974 therapy	[217]
	APCL	BC	High APCL expression correlates with sensitivity to Panobinostat therapy	[296]

*Abbreviations:PDAC* pancreatic ductal adenocarcinoma,*EC* esophageal cancer,*GC* gastric cancer,*CRC* colorectal cancer,*OC* ovarian cancer,*GC* gastric cancer,*BC* breast cancer,*SCLC* small cell lung cancer,*OSCC* oral squamous cell carcinoma,*OS* osteosarcoma,*EMT* epithelial-mesenchymal transition,*LRP* low-density lipoprotein receptor-related protein,*EC* endometrial cancer,*GBM* Glioblastoma multiforme

#### Prognostic biomarkers

Numerous studies have demonstrated that core components of the WNT signaling pathway play a significant role in tumor progression. Abnormal expression of WNT, FZD, β-catenin, and other elements is positively correlated with poor prognosis in various cancers. The WNT signaling pathway holds potential as a prognostic biomarker. For instance, high expression of β-catenin in CC tissues is significantly associated with tumor metastasis, reduced progression-free survival, and overall survival [323]. Additionally, researchers have explored the value of E-cadherin and β-catenin as prognostic biomarkers in esophageal squamous cell carcinoma, revealing that patients with dual-positive expression have significantly higher survival rates compared to those with single-positive or dual-negative expression [331]. In PDAC, β-catenin is significantly upregulated in metastatic tissues, potentially driving metastasis by promoting EMT and matrix metalloproteinase activity.β-catenin specifically upregulated in metastatic lesions may serve as predictive markers for metastasis [315]. Beyond β-catenin, WNT5A also exhibits potential prognostic value in high-grade serous OC, CRC, PCa, EC, and HCC [314, 318, 320, 321, 332]. For example, the expression rate of WNT5A in HCC tissues is significantly lower than in adjacent tissues, and its expression intensity gradually decreases with clinical stage progression. Low WNT5A expression is significantly correlated with poor prognostic factors, including poorly differentiated tumors, portal vein tumor thrombus, high TNM staging, and reduced 5-year survival rates [314]. Conversely, WNT5A is highly expressed in PCa and CRC [320, 332]. In PCa, WNT5A expression is associated with shorter overall survival, higher Gleason scores, and shorter recurrence times. WNT5A-S is highly expressed in CRC cell lines and tumor tissues, while WNT5A-L is significantly low. WNT5A-S has a pro-cancer effect: knocking down its expression can inhibit CRC cell proliferation and induce apoptosis. WNT5A-L has a tumor-suppressive effect: restoring its expression can enhance apoptosis and inhibit β-catenin expression. The combination of high WNT5A-S expression and low WNT5A-L expression is associated with poor patient prognosis [320]. Moreover, WNT8B has also been reported as a potential prognostic biomarker in HCC. High WNT8B expression is linked to adverse pathological features such as large tumor volume, liver cirrhosis, and high ALT levels. Patients with high WNT8B expression have significantly shorter overall survival and recurrence-free survival [317]. Tulu Hong et al. report that BC tissues exhibit high FZD2 expression, which is closely related to poor distant metastasis-free survival and overall survival [333]. Additionally, FZD2 has the potential to predict patient prognosis in other cancers such as serous OC, HCC, and EC [334–336]. Other FZDs, such as FZD3 and FZD6, have been reported as potential prognostic biomarkers for melanoma and OSCC, respectively [198, 337]. TCF7 is a novel independent prognostic biomarker in GC. High TCF7 expression is closely associated with the invasiveness and metastasis of GC, and patients with high TCF7 expression have significantly lower 5-year survival rates compared to those with low expression [319].

#### Diagnostic biomarkers

Identifying clinically valuable tumor biomarkers for precise diagnosis is an auxiliary method to achieve ideal clinical outcomes. Researchers have investigated the diagnostic value of WNT11 in SCLC. WNT11 is involved in regulating neuroendocrine differentiation, cell proliferation, and E-cadherin expression in SCLC, suggesting its close association with the malignant phenotype of SCLC. The high expression of WNT11 in the cytoplasm and perinuclear regions of SCLC cells can serve as a pathological diagnostic marker to distinguish SCLC from NSCLC [324]. Other studies have found that WNT3A can promote malignant transformation of hepatocytes by activating the WNT/β-catenin pathway. WNT3A is significantly upregulated from the early stages of HCC development, and its expression level is positively correlated with disease progression, the sensitivity of which is even superior to AFP. As a dynamically upregulated molecule during HCC development, WNT3A exhibits high sensitivity and specificity, making it a highly promising biomarker for early diagnosis [325]. In OS patients, serum WNT6 levels are significantly higher than those in ES, osteomyelitis patients, and healthy controls. Serum WNT6 may serve as a diagnostic tool for liquid biopsy to differentiate OS from other bone-related diseases. In neuroblastoma, WNT6 is highly expressed in high-risk neuroblastoma without MYCN amplification, potentially serving as a diagnostic marker for this high-risk subtype [338]. Additionally, β-catenin is also a potential diagnostic biomarker. β-catenin is highly expressed in non-seminomatous germ cell tumors but shows very low expression in seminomas. High expression of β-catenin is also associated with high PD-L1 expression and low SII in tumor cells. β-catenin and LEF1 have been used in combined diagnosis. β-catenin is highly expressed in desmoid-type fibromatosis (DF) but shows low sensitivity in basal cell adenoma (BA), whereas LEF1 has significantly higher sensitivity in BA than β-catenin but lower sensitivity in DF. LEF1 can complement β-catenin as a diagnostic marker, and their combined use significantly improves diagnostic accuracy [326]. In sinonasal glomangiopericytoma (SN-GPC), high nuclear expression of β-catenin and LEF1 is observed. In glomus tumors, LEF1 is negative, while it may be partially positive in some solitary fibrous tumors. Therefore, high expression of β-catenin and LEF1 can be used to distinguish SN-GPC from glomus tumors but cannot differentiate it from some solitary fibrous tumors. Researchers have found that all SN-GPC cases do not express stat6, whereas solitary fibrous tumors show strong positivity for stat6. Stat6 negativity helps exclude solitary fibrous tumors, and the combined detection of β-catenin, LEF1, and stat6 can enhance the specificity of SN-GPC diagnosis [328]. Furthermore, in familial adenomatous polyposis (FAP) screening, germline mutations in the *APC* gene are the primary genetic cause of FAP, with a very high risk of developing CRC. Early screening and intervention for FAP patients and their family members can be achieved by detecting APC mutations. Biallelic APC mutations are an early driving event in most sporadic CRC and can be detected at the adenoma stage, indicating its potential as a molecular marker for early diagnosis [339].

#### Therapeutic biomarkers

In OC patients, PORCN as a key enzyme in WNT ligand secretion shows high expression in ascites samples, indicating abnormal activation of the WNT pathway. Studies have demonstrated that high expression of PORCN in OC cells is significantly associated with sensitivity to WNT974, suggesting its potential use in identifying patient populations suitable for WNT974 treatment [217]. In BC, APCl is a tumor suppressor gene and inhibits the WNT/β-catenin signaling pathway by promoting the ubiquitination and degradation of β-catenin. Its upregulation is a crucial mechanism of Panobinostat's therapeutic effect in BC. Kaplan–Meier analysis reveals that high expression of APCL is significantly correlated with better prognosis in BC patients, particularly in Luminal A, Luminal B, and triple-negative subtypes. The expression level of APCL is closely related to the efficacy of Panobinostat and patient outcomes, highlighting its potential as a biomarker for selecting patients for Panobinostat treatment [269].

### Rational combinations of WNT-targeted therapy with other therapeutic strategies

Cancer is an extremely complex disease, and its development involves a series of multiple consecutive mutations in cells. Additionally, chemotherapy can select for mutations that favor the survival of tumor cells, making drug resistance a significant challenge in cancer treatment. Therefore, when targeting the WNT signaling pathway for therapy, single-pathway inhibition may not be sufficient to induce tumor regression. Furthermore, the adverse effects such as gastrointestinal toxicity and fractures observed in clinical trials of WNT pathway inhibitors emphasize that combining WNT-targeted therapy with other treatment modalities is a promising approach to enhance anti-tumor efficacy and reduce cytotoxicity.

Single-pathway targeted therapies often have limited efficacy due to compensatory activation of other pathways, leading to tumor resistance or recurrence. Therefore, combination therapeutic strategies targeting pathway intersections hold greater potential. For instance, the WNT, Hedgehog, and Notch signaling pathways, which are highly conserved, play critical roles in cancer initiation, progression, and recurrence. These pathways not only independently drive tumor formation but also establish a complex cross-regulatory signaling network that maintains CSC stemness and tumor heterogeneity. Consequently, simultaneous inhibition of WNT and Hedgehog or WNT and Notch can synergistically suppress tumor cell proliferation and eliminate CSCs, thereby reducing the risk of recurrence [340]. In GBM, both the PI3K/AKT/mTOR and WNT/β-Catenin pathways are aberrantly activated, regulating cell proliferation, metabolism, and the maintenance of stemness, invasion, and chemoresistance. Preclinical studies have shown that combined use of mTOR inhibitors Rapamycin and WNT pathway antagonists β-catenin siRNA synergistically blocks tumor proliferation, stemness, and drug resistance [341]. Additionally, in AML, the AKT/mTOR and WNT/β-Catenin signaling pathways are hyperactivated in PRL-3-high cancer cells. The combined use of PI3K/mTOR inhibitors VS-5584 and WNT inhibitors ICG-001 has demonstrated significant synergistic cytotoxic effects, inducing apoptosis in PRL-3-high AML cells [342]. Furthermore, in CRC cells with concurrent WNT pathway activation and KRAS mutations, the combination of β-catenin inhibitors PKF115-584 or pyrvinium pamoate and KRAS inhibitors FTS synergistically inhibits cell proliferation and induces apoptosis [343]. Beyond cancer, in non-neoplastic diseases such as ankylosing spondylitis, activation of the WNT and SMAD pathways enhances the osteogenic differentiation capacity of fibroblasts. The combined use of DKK-1 and SIS3, a selective small-molecule inhibitor of SMAD3 that inhibits SMAD3 phosphorylation and attenuates its binding to target DNA, significantly outperforms single inhibitors in suppressing fibroblast proliferation, promoting apoptosis, and inhibiting osteogenic differentiation [344].

The combination of WNT signaling inhibitors with radiotherapy and chemotherapy is also a joint therapeutic strategy. For instance, in CRC, β-catenin is involved in cancer cell resistance to 5-Fluorouracil (5-FU). 4-O-Methylascochlorin effectively inhibits the viability of 5-FU-resistant cells and reduces drug resistance by downregulating the expression of β-catenin. When 4-O-Methylascochlorin is used in combination with 5-FU, it significantly enhances the apoptosis of CRC cells, with a superior effect compared to monotherapy [345]. Additionally, carbon dots (A-CDs) with intrinsic WNT/β-catenin inhibitory capabilities are successfully prepared through a one-step hydrothermal method using aloe. These A-CDs are then used as carriers to load the chemotherapeutic drug 5-FU, forming CDs@5-FU complexes, which are further modified with hyaluronic acid (HA) to achieve tumor targeting (HA-CDs@5-FU). The result shows that HA-CDs@5-FU significantly outperform free 5-FU in inhibiting colon and LCA cells, with lower toxicity to normal liver cells [346]. Niclosamide enhances the binding of the β-catenin destruction complex to β-catenin, promoting its phosphorylation and subsequent ubiquitination, leading to β-catenin degradation via the proteasome, thereby inhibiting the expression of downstream target genes of the WNT/β-catenin pathway. The combination of niclosamide with gemcitabine significantly enhances the inhibition of PCA cell proliferation, outperforming gemcitabine monotherapy [347]. Furthermore, the combination of ICG-001 with radiotherapy significantly inhibits HCC tumor growth and extends the survival period in mice [348].

Many studies have reported the therapeutic effects of combining WNT signaling inhibitors with immunotherapy. For instance, the RNAi drug DCR-BCAT, which targets β-catenin, has been shown to significantly inhibit the growth of various types of tumors when used in combination with anti-PD-1/CTLA-4 antibodies [288]. Clofazimine, by inhibiting the WNT6-mediated WNT/β-catenin signaling pathway, reduces the expression of PD-L1 in tumor cells and suppresses EMT, thereby weakening the tumor's immune escape capability and enhancing the efficacy of anti-PD-1 immunotherapy. The combination of clofazimine and anti-PD-1 has been found to significantly inhibit the proliferation, invasion, and intracranial spread of GBM cells [349]. Additionally, arsenic trioxide has demonstrated significant anti-tumor activity in MCL. The primary mechanisms include the inhibition of the WNT signaling pathway and the suppression of DNA methyltransferase-1 expression, which reactivates silenced tumor suppressor genes or WNT pathway inhibitors through demethylation, further inhibiting tumorigenesis. The combination of arsenic trioxide with the demethylating agent 5-azacytidine has emerged as a potential therapeutic strategy for MCL [350]. Furthermore, a novel WNT signaling inhibitor, Rh-1, has also been reported to exhibit anti-cancer effects when used in combination with PD-1 inhibitors [351].

Inhibitors of the WNT signaling pathway combined with other methods: Researchers investigated the effects of combining Sorafenib and the β-catenin inhibitor FH535 on the metabolism and mitochondrial function of HCC cells. The result shows that Sorafenib alone reduces the glycolytic capacity of HCC cells, while FH535 has no significant effect on this. However, the combination of the two significantly decreases the mitochondrial respiration rate and ATP production, and induces the loss of mitochondrial membrane potential (△Ym), ultimately leading to apoptosis of HCC cells. The synergistic effect of Sorafenib and FH535 stems from the dual inhibition of key metabolic pathways in HCC cells, providing a new approach for the treatment of HCC [352]. In another study, researchers combine targeting the WNT signaling pathway with autophagy inhibition for the treatment of TNBC. They use FZD7-NS in combination with the autophagy inhibitor chloroquine (CQ). FZD7-NS significantly inhibits the expression of WNT downstream genes by blocking the interaction between WNT ligands and the FZD7 receptor, while upregulating the autophagy marker LC3. The combination of FZD7-NS and CQ synergistically reduces the expression of genes associated with CSCs and simultaneously inhibit the migration and self-renewal abilities of BC cells [353].

## Conclusions and perspectives

Despite extensive and prolonged research on this fundamental and highly conserved WNT signaling pathway, we still lack definitive answers on how to safely target it for therapeutic purposes. Over the past 15 years, inhibitors targeting various components of the WNT pathway have shown promising results in preclinical studies, yet no FDA-approved drugs targeting WNT are currently available for clinical use. Acceptable safety remains a central challenge in WNT-targeted therapies, and determining how to selectively target WNT components in specific tumor types while optimizing dosage is key to overcoming current limitations.

Nevertheless, given the substantial body of early research demonstrating the therapeutic potential of WNT modulation in cancer—along with the ongoing discovery of new regulatory factors and pathway components—further investigation into WNT-targeted cancer therapies remains highly warranted. Some researchers have proposed shifting the focus of WNT inhibitor development from upstream WNT effectors to specific mutations in cytoplasmic components to improve specificity. Additionally, they suggest that nanoparticle drug delivery systems may offer a novel approach to cancer treatment due to their potential for targeted cell delivery and precise drug release. Others have highlighted that identifying druggable structures and binding sites within WNT components could facilitate the development of more specific therapeutics. Additionally, to optimize cancer therapeutics targeting the WNT signaling pathway, we also propose several promising research directions, including bispecific antibody development, organ-specific pathway mapping and microenvironment-adaptive delivery systems. These approaches demonstrate significant potential for advancing precision oncology while maintaining fidelity to biological mechanisms.

Through this review, we illustrate the cancer-promoting mechanisms of the WNT signaling pathway from different perspectives and comprehensively review the current research status of WNT signaling pathway inhibitors. Finally, we focus on the possible research directions for optimizing WNT signaling pathway inhibitors, hoping to provide new research ideas for future WNT signaling pathway-targeted therapies.

## Data Availability

Not applicable.

## Abbreviations

PCP: Planar cell polarity
FZD: Frizzled
LRP: Low-density lipoprotein receptor-related protein
RNF43: Ring finger protein 43
ZNRF3: Zinc and ring finger 3
CK1: Casein kinase 1
Ser: Serine
Thr: Threonine
GSK-3β: Glycogen synthase kinase-3β
APC: Adenomatous polyposis coli
PP2A: Protein phosphatase 2A
WTX: Wilms tumor gene on the X chromosome
β-TrCP: β-Transducin repeat-containing protein
CTBP: C-terminal binding protein
GRG: Groucho
HDACs: Histone deacetylases
DVL: Dishevelled
JNK2: Jun N-terminal kinase 2
CaMKII: Calcium calmodulin mediated kinase II
NFAT: Nuclear factor of activated T cells
VANGL: Vang-like
CELSR: Cadherin EGF LAG seven-pass G-type receptor
INTU: Inturned
INVS: Inversin
RYK: Receptor-like tyrosine kinase
MUSK: Muscle-skeletal receptor Tyr kinase
PTK7: Protein tyrosine kinase 7
ROR1/2: Receptor Tyr kinase-like orphan receptor 1/2
DAAM: Dvl-associated activator of morphogenesis
RHOA: Ras homolog family member A
ROCK: RHO-associated coiled-coil-containing protein kinase
DIA1: Diaphanous 1
MRLC: Mitogen-activated protein kinase
CapZIP: CapZ-interacting protein
PLC: Phospholipase C
IP3: Inositol 1,4,5-triphosphate
DAG: 1,2-Diacylglycerol
CaN: Calcineurin
PKC: Protein kinase C
EMT: Epithelial-mesenchymal transition
TGF-β: Transforming growth factor-β
YAP: Yes-associated protein
TAZ: Transcriptional co-activator with PDZ-binding motif
CLL: Chronic lymphocytic leukemia
OSCC: Oral squamous cell carcinoma
JAG1: Jagged 1
NF-κB: Nuclear factor-κB
EDA: Ectodysplasin A
DDX: DEAD-box helicase 5
AURKAIP1: Aurora-A kinase interacting protein 1
CRC: Colorectal cancer
GBM: Glioblastoma multiforme
HCC: Hepatocellular carcinoma
AFP: Alpha-Fetoprotein
AR: Androgen receptor
MSC: Mesenchymal stem cell
NSCLC: Non-small cell lung cancer
CSC: Cancer stem cell
TMD: Transmembrane domain
TNBC: Triple-negative breast cancer
CRD: Cysteine-rich domain
TcdB: Clostridium difficile toxin B
HB: Hepatoblastoma
TNKS: Tankyrase
PARP: Poly(ADP-ribose) polymerase
OS: Osteosarcoma
LDA: Liquidambaric acid
4βHWE: 4β-hydroxywithanolide E
BCL9: B-cell lymphoma 9
CBP: CREB-binding protein
AMP: Adenosine monophosphate
AMPK: Adenosine monophosphate-activated protein kinase
5-LO: 5-lipoxygenase
ADC: Antibody-Drug Conjugation
ASO: Antisense Oligonucleotide
PROTAC: Proteolysis Targeting Chimera
MTD: Maximum tolerated dose
DLT: Dose-limiting toxicities
AML: Acute myeloid leukemia
BTC: Biliary tract cancer
DLBCL: Diffuse large B-cell lymphoma
SCLC: Small cell lung cancer
DF: Desmoid-type fibromatosis
BA: Basal cell adenoma
SN-GPC: Sinonasal glomangiopericytoma
5-FU: 5-Fluorouracil
HA: Hyaluronic acid
CQ: Chloroquine
Asn: Asparagine
CD: Carbon dots
SLL: Small Lymphocytic Lymphoma
OC: Ovarian cancer
GC: Gastric cancer
BC: Breast cancer
EC: Esophageal cancer
PDAC: Pancreatic ductal adenocarcinoma
FL: Follicular lymphoma
MCL: Mantle cell lymphoma
ES: Ewing sarcoma
NHL: Non-Hodgkin lymphoma
GA: Gastric adenocarcinoma
SCC: Squamous cell carcinoma
HL: Hodgkin's lymphoma
STK3: Serine/threonine kinase 3
BMSC: Bone marrow stromal cell
MaSC: Mammary stem cell
ICD: Intracellular domain
PTC: Patched
SMO: Smoothened
PORCN: Porcupine
RSPO: Spondin
CRBN: Cereblon
TRAF2: Tumor necrosis factor receptor-associated factor 2
CAX: Cycloatrobiloxanthone
KRT19: Keratin 19
CC: Cervical cancer
EC: Endometrial cancer
PCa: Prostate cancer
RCC: Renal cell carcinoma
LCA: Lung cancer
PCA: Pancreatic cancer
PD: Programmed death
PK: Pharmacokinetics
mCRC: Metastatic CRC
BRAF: Raf proto-oncogene
HER2: Human epidermal growth factor receptor 2
HR: Hormone receptor
OXA: Oxaliplatin
CD44v6: CD44 containing variable exon 6
CRD-BP: Coding region determinant-binding protein
GEC: Gastroesophageal cancer
